# Single‐Cell Multi‐omics Assessment of Spinal Cord Injury Blocking via Cerium‐doped Upconversion Antioxidant Nanoenzymes

**DOI:** 10.1002/advs.202412526

**Published:** 2025-01-09

**Authors:** Ke Wang, Judun Zheng, Ronghai Li, Tianjun Chen, Yanming Ma, Ping Wu, Jianxian Luo, Jingyi Zhu, Weiqiang Lin, Minghai Zhao, Yue Yuan, Wen Ma, Xiumei Lin, Yang Wang, Longqi Liu, Peng Gao, Hongsheng Lin, Chuanyu Liu, Yuhui Liao, Zhisheng Ji

**Affiliations:** ^1^ Department of Orthopedics The First Affiliated Hospital Jinan University Guangzhou 510632 China; ^2^ Molecular Diagnosis and Treatment Center for Infectious Diseases Dermatology Hospital Southern Medical University Guangzhou 510091 China; ^3^ BGI Research Shenzhen 518083 China; ^4^ BGI Research Hangzhou 310030 China; ^5^ Key Laboratory of Biomaterials of Guangdong Higher Education Institutes Department of Biomedical Engineering, Jinan University Guangzhou 510632 China; ^6^ Institute for Engineering Medicine Kunming Medical University Kunming 650500 China; ^7^ College of Life Sciences University of Chinese Academy of Sciences Beijing 100049 China; ^8^ Shanxi Medical University – BGI Collaborative Center for Future Medicine Shanxi Medical University Taiyuan 030001 China

**Keywords:** cerium‐doped nanoenzymes, myelination, oxidative stress monitoring, reactive oxygen species (ROS), spinal cord injury (SCI)

## Abstract

Spinal cord injury (SCI) impairs the central nervous system and induces the myelin‐sheath‐deterioration because of reactive oxygen species (ROS), further hindering the recovery of function. Herein, the simultaneously emergency treatment and dynamic luminescence severity assessment (SETLSA) strategy is designed for SCI based on cerium (Ce)‐doped upconversion antioxidant nanoenzymes (Ce@UCNP‐BCH). Ce@UCNP‐BCH can not only efficiently eliminate the SCI localized ROS, but dynamically monitor the oxidative state in the SCI repair process using a ratiometric luminescence signal. Moreover, the classic basso mouse scale score and immunofluorescence analysis together exhibit that Ce@UCNP‐BCH effectively facilitates the regeneration of spinal cord including myelin sheath, and promotes the functional recovery of SCI mice. Particularly, the study combines snATAC‐eq and snRNA‐seq to reveal the heterogeneity of spinal cord tissue following Ce@UCNP‐BCH treatment. The findings reveal a significant increase in myelinating oligodendrocytes, as well as higher expression of myelination‐related genes, and the study also reveals the gene regulatory dynamics of remyelination after treatment. Besides, the ETLSA strategy synergistically boosts ROS consumption through the superoxide dismutase (SOD)‐related pathways after SOD‐siRNA treatment. In conclusion, this SETLSA strategy with simultaneously blocking and dynamic monitoring oxidative stress has enriched the toolkit for promoting SCI repair.

## Graphical abstract

The study's innovations include nanoparticles that mimic antioxidants to reduce oxidative stress in spinal cord injuries (SCI). A ratiometric fluorescence probe enables real‐time monitoring of oxidative stress, while Ce@UCNP‐BCH nanoenzymes allow precise redox state tracking. Single‐cell sequencing shows enhanced neuronal regeneration, and liquid chromatography‐mass spectrometry confirms that Ce@UCNP‐BCH promotes recovery through the SOD2 pathway.

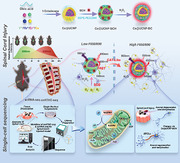

## 1 Introduction

Spinal cord injury (SCI) is a severe disorder of the central nerve system associated with long treatment time, high health‐care costs, high mortality, and disability rate.^[^ ^1^ ^]^ SCI, including primary and secondary injury, has a complex mechanism, thus limiting its recovery.^[^ ^2^ ^]^ However, oxidative stress, characterized by the overproduction of reactive oxygen species (ROS), plays a key role in the aggravation of SCI.^[^ ^3^ ^]^ As a result, alleviating oxidative stress has been widely seen as a promising strategy to attenuate secondary injury and enhance SCI recovery.^[^ ^3^, ^4^ ^]^ Recently, nanotechnology possessing ROS‐eliminate ability has received much attention for its precise drug delivery and enhanced retention effect to obtain optimal therapeutic benefits^[^ ^5^ ^]^ Specifically, nanozymes are a class of nanomaterial‐based artificial enzymes, and have been applied in the treatment of ROS‐related neurological diseases and neuroprotection own to penetrate the blood‐spinal cord barrier (BSCB).^[^ ^6^ ^]^ Among them, CeO_2_ nanoparticles (CeO₂ NPs) have been proven to simulate endogenous antioxidants, such as superoxide dismutase (SOD) and nitric oxide scavenging activity, thus eliminating the excessive ROS or RNS.^[^ ^7^ ^]^ Although these materials can achieve a considerable therapeutic effect by alleviating oxidative stress, the extent of oxidative stress at the site of SCI cannot be simultaneously monitored, let alone real‐timely adjusting the dose of drugs. Therefore, simultaneously blocking and dynamic monitoring the oxidative stress state in SCI is urgent and meaningful.

Several imaging technologies, such as computed tomography, magnetic resonance imaging, and positron emission tomography, have been developed for the diagnosis and prognosis of SCI,^[^ ^8^ ^]^ However, the pathological changes and screen the degree of oxidative stress cannot be precisely realized. Recent advances in the responsive fluorescent probes have shown the superiority toward distinguishing the SCI redox state due to their higher selectivity and sensitivity.^[^ ^9^ ^]^ But most fluorescent probes cannot achieve synchronous monitoring of reducing and oxidizing molecules using one signal, which was susceptible to some interferences including probe concentration, photo‐bleaching, and solvent.^[^ ^10^ ^]^ Thus, the ratiometric fluorescence sensors have been developed and applied to the real‐time monitoring of specific molecule markers,^[^ ^11^ ^]^ providing a reliable and accurate analysis method. Particularly, ratiometric probes combining rare‐earth metal‐doped upconversion nanoparticles (UCNPs) and organic molecules were developed based on luminescence resonance energy transfer (LRET),^[^ ^12^ ^]^ where a specific target‐recognizing dye acts as the energy acceptor, thus quenching luminescence of UCNPs and providing turn‐on signals.^[^ ^13^ ^]^ However, UCNPs alone cannot achieve real‐time monitoring of the redox status of intracellular or injured tissue sites, necessitating its modification. Some UCNPs‐based nanocomposites have been developed based on LRET, where a specific target recognizing probe or dye acts as the energy acceptor, thus quenching luminescence of UCNPs and leading to the emission of another light wavelength. Recent studies have shown that UCNPs‐based nanocomposites can be used to monitor intracellular ROS, such as H_2_O_2_, ONOO^−^, and •OH.^[^ ^14^ ^]^ Nonetheless, these nanoprobes cannot synchronously monitor the change of redox state in SCI.

**Scheme 1:**
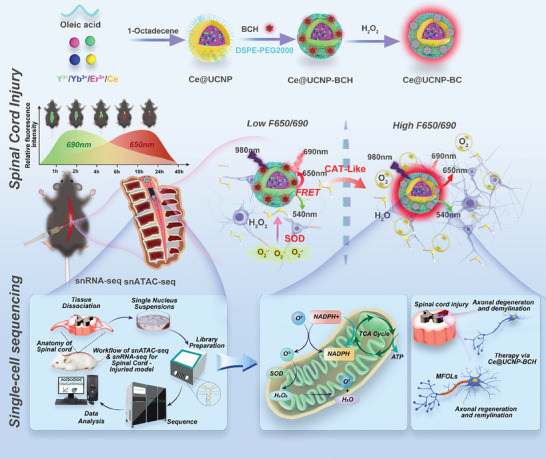
Ameliorating SCI via Ce@UCNP‐BCH promotes real‐time luminescence and single‐cell multi‐omics evaluation.

Herein, the simultaneously emergency treatment and dynamic luminescence severity assessment (SETLSA) strategy were developed based on the cerium (Ce)‐doped upconversion antioxidant nanoenzymes (Ce@UCNP‐BCH). This strategy can consume ROS and simultaneously monitor the degree of oxidative stress in real‐time, achieving adapting SCI treatment. The upconversion luminescence (UCL) of Ce@UCNP‐BCH at 650 nm was quenched under physiological condition when excited by 980 nm near‐infrared ray, while the 690‐nm fluorescence of BCH was emitted based on the LRET. In contrast, the 690 nm light was quenched under excess ROS, while UCL at 650 nm was preserved (**Scheme** **1**). This study first aimed to evaluate the effect of alleviating oxidative stress of Ce@UCNP‐BCH nanoenzymes in vitro and in vivo. Notably, Ce@UCNP‐BCH can effectively facilitate the regeneration of spinal cord tissues, including myelin sheath, and significantly improve the mobility of SCI contusion mouse model.

SCI model is complex and highly heterogeneous, and its regenerative difficulties are caused by a combination of cell‐intrinsic factors and the extrinsic injury environment, including neuronal and glial components and extracellular components.^[^ ^15^ ^]^ Moreover, the SCI model was evaluated based on multiple dimensions by combining multi‐omics to improve the understanding of molecular mechanisms and signaling pathways involved in the Ce@UCNP‐BCH‐treated SCI. The development of single‐cell sequencing technology has improved the understanding of the cellular landscape of SCI at single‐cell resolution, and complex molecular mechanisms, intercellular, and cell‐microenvironment communication, thus greatly enhancing understanding of SCI models.^[^ ^15^, ^16^ ^]^ First, spinal cord tissues were extracted from three groups of adult mice (the sham‐operated group, SCI group, and Ce@UCNP‐BCH drug treatment group). The cellular landscape of the spinal cord was then determined via snATAC‐seq and snRNA‐seq analyses. The changes in the abundance of cellular components and gene expression were compared between different conditions. Results showed that Ce@UCNP‐BCH treatment after SCI could promote neuronal regeneration and coordinate oligodendrocytes to promote neuronal axon remyelination, thus enhancing signaling and tissue remodeling. Ce@UCNP‐BCH treatment also promoted the recovery of mitochondrial function after SCI and activated the signaling pathways related to energy supply and metabolism to provide sufficient energy for key regeneration processes, such as cytoskeletal reconstruction, nutrient transport, and axon remodeling. Besides, liquid chromatography‐mass spectrometry and bioinformatics analysis showed that Ce@UCNP‐BCH enhances the function recovery of SCI mouse mainly through SOD2. Herein, a Ce‐doped upconversion nanoenzymes, acting as ratiometric luminescence probe, was developed. This molecule could achieve real‐time evaluation of the severity of SCI and simultaneously eliminate excessive ROS. Therefore, this molecule can guide the dosage and timing of drug administration, enhancing the development of personalized SCI treatment. In summary, the SETLSA strategy provide new valuable research for SCI treatment.

## 2 Results

### 2.1 Synthesis and Characterization of Ce@UCNP‐BCH

**Figure 1:**
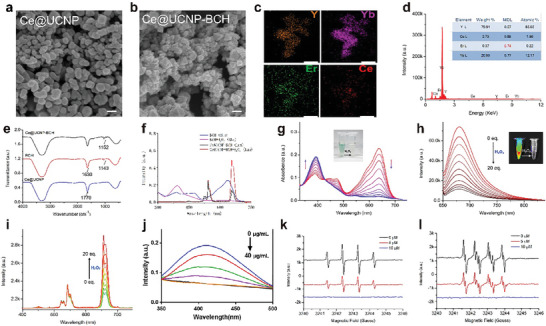
Characterizing data on the Ce@UCNP‐BCH. a) TEM showing Ce@UCNP image (Scale bar = 100 nm). b) TEM showing Ce@UCNP‐BCH image, Scale bar = 100 nm). c) Corresponding elemental mapping images of Ce@UCNP‐BCH. d) EDS spectrum of Ce@UCNP‐BCH. e) Fourier transform infrared spectra of the Ce@UCNP, BCH, and Ce@UCNP‐BCH. f) Absorbance spectrum of BCH in the absence or presence of H_2_O_2_ and luminescence spectrum of Ce@UCNP‐BCH in the absence or presence of H_2_O_2_. g) Absorbance changes of BCH after titration with H_2_O_2_ (0‐20 eq) (inset: visualization of the reaction process). h) Fluorescence intensity changes of Ce@UCNP‐BCH at 690 nm under different concentrations of H_2_O_2_ (0–20 eq) (inset: visualization of the reaction process). i) Fluorescence intensity changes of Ce@UCNP‐BCH at 650 nm under different concentrations of H_2_O_2_ (0–20 eq). j) The antioxidant activity of Ce@UCNP‐BCH based on hydrogen peroxide (H_2_O_2_) scavenging assay. k,l) Electron paramagnetic resonance (EPR) spectra of the H_2_O_2_ and O_2_^•−^ under different concentrations of Ce@UCNP‐BCH.

The SETLSA strategy were developed by loading H_2_O_2_‐responsive probe BCH in the synthesized Ce‐codoped UCNPs, forming Ce@UCNP‐BCH. Transmission electron microscopy (TEM) analysis demonstrated that the Ce@UCNP‐BCH nanoparticles had similar morphology and 100 nm size (**Figure** **1**a,b). Notably, the structure of Ce@UCNP and Ce@UCNP‐BCH nanoparticles was not significantly different. Meanwhile, elemental analysis and X‐ray photoelectron spectroscopy (XPS) showed that yttrium, ytterbium, erbium, and Ce were all distributed in the as‐prepared Ce@UCNP‐BCH nanoparticles (Figure 1c,d). Fourier infrared spectroscopy (FT‐IR) further demonstrated that BCH was successfully loaded in Ce@UCNP (Figure 1e), forming Ce@UCNP‐BCH. Further analysis was performed to assess whether the LRET between UCNPs and BCH, as the energy donor and receptor, was controlled by H_2_O_2_. The absorption peak of BCH showed a significant blue shift from 647 to 414 nm after reaction with H_2_O_2_ (Figure 1f). Meanwhile, the absorbance peak of BCH at 647 nm showed a wide overlap with the emissions of UCL at 650 nm (I_650_) of UCNPs, forming an integrated energy donor and receptor system. Finally, the I_650_ of UCNPs was quenched, and the emission (up to 690 nm) of BCH was sensitized at 647 nm. A more reliable result of H_2_O_2_ monitoring and imaging could be obtained using the ratio of I_690_ to I_650_ as signal intensity, which weakens the background signal interference. The UV‐VIS spectra of BCH and the UCL spectra of Ce@UCNP‐BCH were assessed under different H_2_O_2_ concentrations to evaluate whether Ce@UCNP‐BCH can detect H_2_O_2_ in vitro (Figure 1g–i). The quenching efficiency of BCH toward I_650_ gradually decreased with the addition of hydrogen peroxide, leading to a gradual restoration of the red I_650_ emission. Taken together, these findings indicate that the ratiometric UCL nanoprobes Ce@UCNP‐BCH exhibit good photophysical properties and high sensitivity for H_2_O_2_ monitoring, making it have a broad application prospect in biomedical applications. Moreover, Ce@UCNP‐BCH significantly scavenged the free hydrogen peroxide (H_2_O_2_) in a dose‐dependent manner, indicating that they possess excellent ROS‐scavenging activity (Figure 1j). Additionally, electron paramagnetic resonance (EPR) spectra revealed that Ce@UCNP‐BCH remarkably decreased the characteristic spectrum intensity of H_2_O_2_ and superoxide anion (O_2_^•−^), indicating that the nanozymes effectively scavenged ROS (Figure 1k,l). Meanwhile, the biostability of Ce@UCNP was also evaluated by measuring its luminescence in PBS solution with various conditions containing different serum (from 0 to 100%) or in 100% serum keeping for various time. Stable luminescence of the nanoprobe under these conditions was observed (Figure S1, Supporting Information), which indicated its high stability in the simulated physiological environments.

### 2.2 ROS‐Scavenging Performance and Neuroprotective Ability of CeO_2_ NPs and Ce@UCNP In Vitro

**Figure 2:**
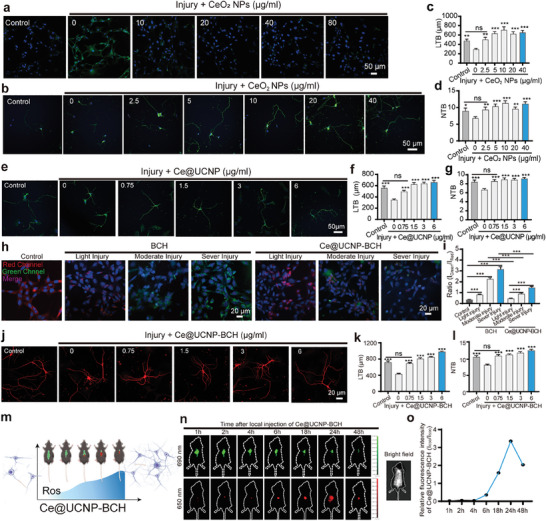
Detection of redox state and restoration of neural behaviors using Ce@UCNP‐BCH. a) Confocal microscopy fluorescence images of neurons stained by DCFH‐DA under various concentrations of CeO_2_ NPs, Scale bar = 50 µm. b) Confocal microscopy fluorescence images of neurons under different concentrations of CeO_2_ NPs, Scale bar = 50 µm. c) Relative length (LTB) and d) the number (NTB) of each branch for b (n = 30). e) Confocal microscopy images of neurons under various concentrations of Ce@UCNP, Scale bar = 50 µm. f) Relative length (LTB) and g) the number (NTB) of each branch for e) (n = 30). h) Fluorescence behaviors of BCH and Ce@UCNP‐BCH in neurons with different degrees of injury. i) Quantified ratios of I_650_/I_690_ intensity of h (n = 6). j) Confocal microscopy fluorescence images of neuronal treatments under various Ce@UCNP‐BCH concentrations, Scale bar = 20 µm. k) Relative length (LTB) and l) the number (NTB) of each branch for j) (n = 30). m) Redox state detection using Ce@UCNP‐BCH. n) UCL imaging of Ce@UCNP‐BCH in vivo. o) Quantified ratios of I_650_/I_690_ intensity of n (n = 5). * means *p* < 0.05, ** means *p* < 0.01, *** means *p* < 0.001, ns means not significant.

The ROS‐scavenging and neuroprotective ability of CeO_2_ NPs and Ce@UCNP in vitro were assessed as follows: First, Cell Counting Kit‐8 (CCK‐8) was used to evaluate the cytotoxicity of CeO_2_ NPs. CCK‐8 showed that the concentration of CeO_2_ NPs below 20 µg mL^−1^ showed no significant toxicity in HT‐22 cell, implying that CeO_2_ NPs have good biocompatibility (Figure S2a, Supporting Information). The intracellular ROS level was investigated via DCFH‐DA (2′‐7′‐dichlorodihydrofluorescein diacetate) assay to estimate the ROS‐scavenging ability of CeO_2_. Results showed that CeO_2_ NPs could significantly decrease the fluorescence intensity of DCF in Glutamic acid‐activated HT‐22 cells, indicating that CeO_2_ NPs can eliminate excessive ROS (**Figure** **2**a; Figure S2b, Supporting Information). The DHE (dihydroethidium) assay, mainly detecting the intracellular O_2_^•−^ level, was also conducted to assess whether CeO_2_ NPs can scavenge O_2_^•−^. Results showed that CeO_2_ NPs can significantly scavenge the intracellular O_2_^•−^ (Figure S2c,d, Supporting Information). Furthermore, flow cytometric analysis showed that CeO_2_ NPs significantly reduced the relative ROS expression in HT‐22 cells (Figure S3, Supporting Information). These results suggest that CeO_2_ NPs can effectively eliminate excessive ROS.

Further analysis investigated whether CeO_2_ NPs can promote the recovery of damaged neurons. Notably, CeO_2_ NPs (below 40 µg mL^−1^) showed no significant cytotoxicity in neurons. Therefore, 40, 20, 10, 5, 2.5, and 0 µg mL^−1^ were selected for the next experiment (Figure S4a, Supporting Information). The post‐injured hippocampal neurons were incubated with the medium containing different CeO_2_ NPs concentrations. The length and number of neuron protrusions were then measured. The findings indicated that CeO_2_ NPs significantly increased the length and quantity of neuron protrusions_,_ especially at 10 µg mL^−1^ (Figure 2b–d; Figure S4b–e, Supporting Information). Furthermore, Ce@UCNP was synthesized by doping Ce into up‐conversion nanoparticles for further analysis. Further analysis was conducted to assess whether Ce@UCNP can eliminate excessive ROS and promote the recovery of damaged neurons. Besides, CCK‐8 showed that Ce@UCNP (below 6 µg mL^−1^) had no cytotoxicity in HT‐22 cells (Figure S5a, Supporting Information). Therefore, 6, 3, 1.5, 0.75, and 0 µg mL^−1^ Ce@UCNP were selected for the next experiments. The DCFH‐DA assay revealed that the fluorescence intensity of DCF significantly decreased in HT‐22 cells treated with Ce@UCNP (Figure S5b,c, Supporting Information). DHE assay also showed that the red fluorescence intensity was significantly reduced in HT‐22 cells after treatment with Ce@UCNP (Figure S5d,e, Supporting Information). Similarly, flow cytometry also showed that the mean fluorescence intensity reduced in HT‐22 cells (Figure S5f,g, Supporting Information). These results indicate that Ce@UCNP can effectively eliminate excessive ROS. Notably, Ce@UCNP showed no significant cytotoxicity in hippocampal neurons below 6 µg/mL (Figure S6a, Supporting Information). Therefore, 6, 3, 1.5, 0.75, and 0 µg mL^−1^ Ce@UCNP were selected for the next experiments. (Figure 2e–g; Figure S6b–e, Supporting Information). These results indicate that Ce@UCNP can significantly enhance the recovery of the damaged neurons.

### 2.3 Fluorescence Behaviors and Neural Restoration Behaviors of Ce@UCNP‐BCH In Vitro

Although results have shown that Ce@UCNP can attenuate the intracellular ROS and promote the recovery of damaged neurons, it cannot monitor the intracellular redox state in real time. Therefore, BCH, fluorescence probes whose absorption peak shifted from 647 nm to 414 nm when H_2_O_2_ was added, was introduced into Ce@UCNP.^[^ ^10a^ ^]^ The shift of absorption peak of BCH in the damaged HT‐22 cells was then assessed. Moreover, CCK‐8 indicated that BCH concentrations below 10 µg mL^−1^ had no cytotoxicity in HT‐22 cells (Figure S7a, Supporting Information). Furthermore, the normal and injured HT‐22 cells were incubated with BCH for further analysis. Compared with the normal cells, the red fluorescence intensity was significantly decreased in the injured cells, while the green fluorescence intensity was significantly increased (Figure S7b, Supporting Information). The fluorescence intensity ratio between the green and the red fluorescence (Figure S7c, Supporting Information) was similar as in Figure S7b (Supporting Information). The fluorescence behaviors of Ce@UCNP‐BCH were also assessed. Notably, Ce@UCNP‐BCH showed no significant cytotoxicity below 24 µg mL^−1^ (Figure S7d, Supporting Information). Therefore, 24 µg mL^−1^ Ce@UCNP‐BCH was selected to investigate the fluorescence behaviors of Ce@UCNP‐BCH. Moreover, the green fluorescence intensity was significantly increased by BCH and Ce@UCNP‐BCH with increasing degree of injury, while the red fluorescence intensity decreased (Figure 2h,i). In addition, to further verify the monitoring capability of BCH probes on Ce@UCNP ROS clearance, we set up four groups for HT‐22 cells (control group, injured group, and Ce@UCNP or Ce@UCNP‐BCH drug treatment group). The control, injured and Ce@UCNP group were each introduced equivalent BCH. Compared with the injured group, Ce@UCNP, and Ce@UCNP‐BCH significantly decreased the green fluorescence intensity while it significantly increased the red fluorescence intensity (Figure S7e, Supporting Information). And the quantitative analysis of fluorescence intensity was similar in control, Ce@UCNP, and Ce@UCNP‐BCH group (Figure S7f, Supporting Information). In conclusion, Ce@UCNP‐BCH shows good ROS dynamic monitoring capability. Further analysis was conducted to assess whether Ce@UCNP‐BCH can promote the recovery of damaged neurons. Ce@UCNP‐BCH concentrations below 6 µg/mL showed no significant cytotoxicity (Figure S8a, Supporting Information). The following results indicated that Ce@UCNP‐BCH can effectively promote the recovery of injured neurons (Figure 2j–l; Figure S8b–e, Supporting Information). Therefore, Ce@UCNP‐BCH can monitor the redox status within cells and enhance the recovery of injured neurons.

### 2.4 Upconversion Luminescence Imaging and ROS‐Scavenging of Ce@UCNP‐BCH In Vivo

This study aimed to evaluate whether Ce@UCNP‐BCH can be used to assess the extent of SCI based on a contusion model by monitoring the redox state of the site of injury. The ROS‐scavenging capacity of Ce@UCNP‐BCH was also evaluated in vivo. The experimental procedure of SCI is shown in Figure S9a (Supporting Information). The ratio of fluorescence intensity at 650 nm to fluorescence intensity at 690 nm (I_650_/I_690_) may indicate the signal for monitoring the redox state based on photophysical characteristics of Ce@UCNP‐BCH. The fluorescence image of the SCI model mouse was detected at various time intervals (1, 2, 4, 6, 18, 24, and 48 hours) post‐injection of Ce@UCNP‐BCH at 650 nm under the excitation of a 980‐nm laser and 690 nm under the excitation of a 660‐nm laser (Figure 2m,n). The results showed that I_650_ _nm_ gradually increased with time, peaking at 24 hours post‐injection while I_690_ _nm_ gradually decreased. The ratio of I_650_/I_690_ also showed that the peaks of I_650_/I_690_ occurred at 24 hours post‐injection (Figure 2o). Moreover, I_690_ gradually disappeared after the intraperitoneal injection of BCH probe, but there was no signal of channel 650 nm (Figure S9b, Supporting Information). However, both signals were not detected when PBS was intraperitoneally injected (Figure S9c, Supporting Information). These results suggest Ce@UCNP‐BCH can be used to monitor the redox status of the injured site and the extent of SCI.

**Figure 3:**
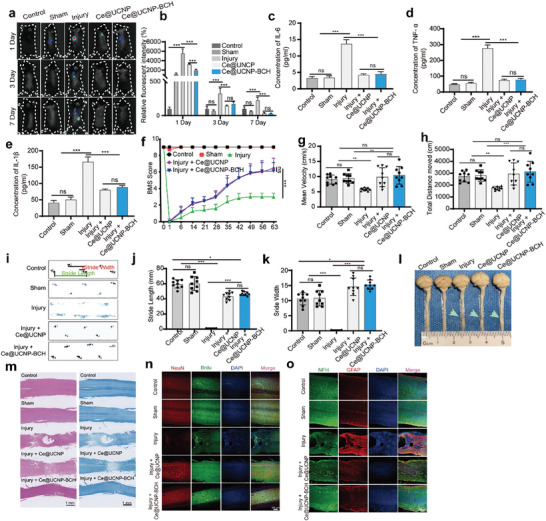
ROS‐scavenging and therapeutic effects of Ce@UCNP‐BCH in vivo. a) Representative images of L‐012‐stained mice after different treatments. b) Quantitative analysis of the intensity of ROS in mice (n = 3). c–e) The expression levels of IL‐6 c, TNF‐α d, and IL‐1β e in the SCI mouse after treatments (n = 3). f) BMS scores of SCI mice in the control, sham, injury, injury + Ce@UCNP, and injury+ Ce@UCNP‐BCH groups. Data are presented as mean ± SD (n = 9). Mean velocity (g) and total distance traveled (h) were used in an open field test to assess the recovery of motor skills in mice (n = 9). i) Mouse footprints from the control, sham, injured, injured + Ce@UCNP, and injured + Ce@UCNP‐BCH groups. j,k) Histograms of the stride length (j) and stride width (k) of mouse in the control, sham, injury, injury + Ce@UCNP, and injury+ Ce@UCNP‐BCH groups (n = 9). l) Representative images of mouse spinal cord tissue in the control, sham, injury, injury + Ce@UCNP, and injury+ Ce@UCNP‐BCH groups. The green arrow points to the site of injury. m) The images showing the location of the damaged spinal cord tissue in each experimental group after being stained with H&E (left) and LFB (right), scale bar=1 mm. n) The fluorescent images showing the spinal cord for immunofluorescence analysis co‐stained with anti‐NeuN antibodies (red) and anti‐BrdU (green), scale bar = 200 µm. o. The fluorescent images showing the spinal cord for immunofluorescence analysis co‐stained with anti‐GFAP antibodies (red) and anti‐NFH (green), scale bar = 200 µm. * means *p* < 0.05, ** means *p* < 0.01, *** means *p* < 0.001, ns indicate not significant.

Previous experiments showed that Ce@UCNP‐BCH can scavenge excessive ROS in vitro. Therefore, further in vivo study was conducted using L‐012, a ROS bioluminescence probe, to detect the ROS level of the site of injured spinal cord tissues.^[^ ^17^ ^]^ The bioluminescence images were recorded at 1, 3, and 7 days post‐SCI. Although no ROS bioluminescence was detected in the control group, ROS bioluminescence was significantly enhanced in the injury group. Furthermore, the intensity of bioluminescence was significantly reduced in the mice treated with Ce@UCNP or Ce@UCNP‐BCH compared with the injury group (**Figure** **3**a,b). These results suggest that Ce@UCNP‐BCH can significantly eliminate excessive ROS in vivo. We then intended to assess the capability of our nanoagent in monitoring the accumulation and distribution behavior of Ce elements in vivo using the inductively coupled plasma mass spectrometry (ICP‐MS) of ex vivo organs. As shown in Figure S10 (Supporting Information), we can still recognize the relatively high level of Ce elements in the spinal cord tissue within 48 h, which was likely due to the PEG coating on the surface of Ce@UCNP, which can delay their macrophage clearance during blood circulation. Meanwhile, blood circulation investigation showed that the blood levels of Ce@UCNP reduced gradually over time but were maintained at a relatively high level even at 12 h postinjection (Figure S11, Supporting Information). The feces and urine of those mice injected with Ce@UCNP were also collected to study its clearance pathway. Intense luminescence at 540 nm was detected in both urine and feces after intraperitoneal injection of Ce@UCNP, indicating that it could be excreted through both renal and fecal, and leaving very little retention of remaining nanoparticles in the mouse body after 7 days (Figure S12, Supporting Information). Previous results also showed that attenuating ROS can regulate the inflammatory response.^[^ ^18^ ^]^ Therefore, the concentration of IL‐1β, IL‐6, IL‐10, and TNF‐α in the serum of the mouse was detected using ELISA analysis for further analysis. The level of IL‐1β, IL‐6, IL‐10, and TNF‐α was significantly increased in the injury group compared with the control and sham group. However, Ce@UCNP or Ce@UCNP‐BCH decreased the level of IL‐1β, IL‐6, IL‐10 and TNF‐α (Figure 3c–e; Figure S13, Supporting Information). In summary, Ce@UCNP‐BCH can effectively attenuate the excessive ROS in the SCI mouse and reduce SCI based on inflammation response.

### 2.5 Therapeutic Effect of Ce@UCNP‐BCH In Vivo

Results above showed that Ce@UCNP‐BCH can promote the recovery of damaged neurons in vitro and can attenuate the excessive ROS in vitro and in vivo. Therefore, the SCI contusion model was established to verify whether Ce@UCNP‐BCH can facilitate the restoration of hind limb functionality of SCI mice. Experimental procedure of Ce@UCNP‐BCH treatment is shown in Figure S14a (Supporting Information). Notably, the weight of all mice was not significantly different among the groups after injury (Figure S14b, Supporting Information). The hindlimb mobility functionality recovery was assessed based on BMS scores. The BMS score was significantly higher in the Ce@UCNP‐BCH and Ce@UCNP groups than in the injury group (Figure 3f). The functionality of the hindlimb motion was also accessed via OFT. Compared with the injury group, the mean maximum velocity, and total distance moved by the mouse in the Ce@UCNP‐BCH and Ce@UCNP group were significantly increased (Figure 3g,h; Figure S14c, Supporting Information). The gait of SCI mice was assessed using footprint test. The typical footprint images from every group are shown in Figure 3i. Compared with the injury group, the hindlimb motor function of the Ce@UCNP‐BCH or Ce@UCNP group mice had considerably enhanced stride length (Figure 3j) and stride breadth (Figure 3k). The hindlimb motor function was also assessed using catwalk test. Compared with the injury group, hind max contact, and regularity index were significantly enhanced in the Ce@UCNP‐BCH and Ce@UCNP groups (Figure S10d–f, Supporting Information). These results indicate that Ce@UCNP‐BCH can effectively promote hindlimb mobility functionality recovery in vivo.

The therapeutic effects of Ce@UCNP‐BCH were also assessed through pathological analysis of the tissue in the spinal cord. First, the biological safety of Ce@UCNP‐BCH was assessed as follows: The primary organs were harvested from each group, followed by H&E staining. Results showed no significant changes in the organs in all groups (Figure S15, Supporting Information). The spinal cord was then harvested from all groups for H&E staining, LFB staining, and immunostaining analysis (Figure 3l,m). Compared with the control and sham groups, the morphological integrity of the spinal cord site was significantly disrupted in the injury group, and the scar significantly shrank. Ce@UCNP‐BCH significantly improved the spinal cord tissue. H&E staining showed similar results. The extent of myelin injury and axonal demyelination was analyzed via LFB staining. Ce@UCNP‐BCH reduced demyelination to protect nerve fibers since the preservation of sheaths considerably improved in the Ce@UCNP‐BCH group than in the injury group. The protective therapeutic effect of Ce@UCNP‐BCH was verified via tunnel staining. The number of tunnel‐positive cells was lower in the Ce@UCNP‐BCH group than in the injury group (Figure S16a, Supporting Information). These results indicate that Ce@UCNP‐BCH can significantly reduce the apoptosis at spinal cord injury. Furthermore, the function and mechanism of Ce@UCNP‐BCH on injured spinal cord tissue repair were assessed via NeuN/Brdu co‐staining. BrdU incorporation into replicating DNA allows for the evaluation of cellular proliferative capacity, as it substitutes for thymidine and is passed on to daughter cells.^[^ ^19^ ^]^ NeuN, primarily expressed in central nervous system cells, serves as an effective marker for neuronal identification.^[^ ^20^ ^]^ The incidence of neuron loss was higher in the injury group than in other groups, indicated by the decreased number of NeuN‐positive cells (Figure 3n). However, the number of cells in the Ce@UCNP‐BCH group expressing NeuN increased (similar to the control or sham group), implying that Ce@UCNP‐BCH can promote the survival of neurons in the injured spinal cord tissues. In addition, the number of NeuN and Brdu positive cells increased in the Ce@UCNP‐BCH group compared with the injury group. Furthermore, more nestin‐positive cells were detected in the Ce@UCNP‐BCH group than in the injury, sham, and control groups (Figure S16b, Supporting Information). The excessive formation of glial scar can impede the growth and recovery of axons, and hinder survival and regeneration. Consequently, NFH/GFAP co‐staining was processed to elucidate whether Ce@UCNP‐BCH can regulate glial scar formation, thus promoting neuron regeneration. Compared with the injury group, NFH intensity significantly increased in the Ce@UCNP‐BCH group, while GFAP formation decreased around the injured site of the spinal cord (Figure 3o), implying that Ce@UCNP‐BCH can enhance the regeneration of neurons by inhibiting glial scar formation. These results indicate that Ce@UCNP‐BCH can promote hindlimb movement function recovery of SCI mice and the regeneration of injured spinal cord tissue by promoting the survival of neurons and inhibiting glial scar formation.

Furthermore, In order to validate the repair of BSCB, endothelial cells were first chosen as the transwell cell model, because BSCB is mainly composed of endothelial cells, pericytes, and astrocyte, all of them work together to preserve the chemical components of the neural environment to keep the brain or spine functioning normally.^[^ ^21^ ^]^ Before the assessment of the barrier repair condition, the protective effects of the nanoenzymes‐treated mouse brain microvascular endothelial cell line (bEnd.3) against oxidative stress injury was analyzed by apoptosis staining kit and flow cytometric counting. As shown in the Figure S17 (Supporting Information), the apoptosis ratio of hydrogen peroxide (H_2_O_2_)‐pretreated bEnd.3 cell group incubated with CeO_2_ NPs or Ce@UCNP were significantly reduced compared with the PBS‐treated control group, demonstrating the CeO_2_ NPs and Ce@UCNP were able to prevent cells from undergoing oxidative damage. Next, the repair effect of nanoenzymes on the BSCB function were verified via transwell permeation assay. As shown in Figure S18a (Supporting Information), we constructed the BSCB tissue model by planting bEnd.3 cells on the upper chamber, and then H_2_O_2_ was added into the above chamber for obtaining the oxidative stress‐damage BSCB model. Subsequently, the oxidative stress‐damage BSCB group was respectively treated with CeO_2_ NPs and Ce@UCNP or PBS, and the fluorescence intensity of FITC‐Dextran in the lower chamber was analyzed by microplate reader. As indicated in Figure S18b (Supporting Information), the fluorescence intensity of the control group was significantly lower compared to the PBS‐treated groups, indicating the successful construction of oxidative stress‐damage BSCB model. Conversely, the fluorescence intensity in both the CeO_2_‐treated and Ce@UCNP‐treated groups was comparable to that of the control group, suggesting a notable repair effect of nanoenzymes treatment. Meanwhile, the above nanoenzyme‐treated bEnd.3 cells were stained by immunofluorescence, and the morphology and number of endothelial cells were assessed (Figure S19, Supporting Information). The number of bEnd.3 cells displayed more numerous than the injury group, following with the gradual recovery of the morphology, which was in accordance with these previous results. Furthermore, the intensity of FITC‐Dextran fluorescence was significantly reduced in the SCI site treated with CeO_2_ NPs or Ce@UCNP compared with the injury group (Figure S20, Supporting Information). These results suggest that Ce@UCNP can significantly repair the BSCB in vivo. Taken together, these results firmly indicated that the antioxidant property of our Ce@UCNP can effectively facilitate the recovery of BSCB function, revealing the feasibility of our nanoprobe for further improving the mobility of SCI mice.

### 2.6 Comparative Analysis of Cellular and Molecular Changes after SCI and Treatment

**Figure 4:**
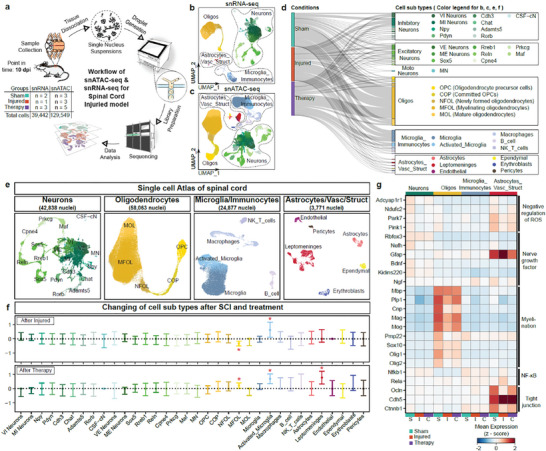
Single cell Atlas of spinal cord and differences in cell type and gene expression between conditions. a) Overview of this study, n represents biological replicates. b) UMAP plot showed 39442 spinal cord nuclei of snRNA‐seq. c) UMAP plot showed 129 549 spinal cord nuclei of snATAC‐seq. d) Sankey diagram showed the percentage of cell subtypes in different conditions in snATAC‐seq. e) UMAP plot showed main cell types of snATAC‐seq, including Neurons, Oligodendrocytes, Microglia & Immunocytes, Astrocytes & Vascular & Structured cells. f) The error bar plot illustrates the changes in cell subtypes following injury or Ce@UCNP‐BCH therapy, as analyzed using scCODA. Positive and negative values represent increases and decreases in cell abundance, respectively, with red asterisks indicating statistically significant changes (FDR = 0.05). Sample sizes for the analysis were: Sham group (n = 3), Injured group (n = 3), and Therapy group (n = 5). g) Heat map with additional information on main cell type (top) and condition (bottom) showing z‐scores of normalized, log‐transformed, and scaled expressions of main genes of selected pathways in snRNA‐seq.

Previous experimental results have demonstrated the overall efficacy of Ce@UCNP‐BCH in the treatment of SCI. This includes the promotion of spinal cord repair, improvement of motor function, and the clearance of ROS. Nevertheless, following a SCI, in situ heterogeneity within tissues is increased. The cellular subpopulations present may exhibit differential responses and recovery capabilities. In order to elucidate the precise mechanism of action of Ce@UCNP‐BCH, mice from the sham, injury, and therapy groups were used to collect spinal cords in the subacute phase (10 dpi) for snATAC‐seq and snRNA‐seq (BGI DNBelab C4) (**Figure** **4**a). For data quality control, we implemented a standard data pre‐processing procedure (Figure S21a, Supporting Information). Finally, 129549 cells were obtained in snATAC‐seq and 39442 cells in snRNA‐seq after strict quality control and filtering (Figure S21b,c, Supporting Information). For cell annotation, the major cell types of the snRNA dataset were first defined using the marker genes from the public dataset.^[^ ^16^ ^]^ (Figure 4b; Figure S21d, Supporting Information). We carefully defined each cell subtype using marker genes, with integration of the highly expressed genes in each cluster (Figure S22a, Supporting Information). The same strategy was used to define cell types in the snATAC dataset (Figure 4c). We manually grouped it into subsets, then annotated by the snRNA dataset using label transfer. Jaccard indices between annotations were manually determined, and annotation was achieved by label transfer, resulting in a high correspondence (Figure S22b, Supporting Information). It is noteworthy that, in comparison to snRNA‐seq, we defined two additional cell types in snATAC‐seq: one for erythroblasts and one for pericytes (Figure S22b,c, Supporting Information). This indicates that snATAC‐seq and snRNA‐seq are mutually reinforcing in the identification of cell types and can identify and annotate different cell types in a more comprehensive manner. Finally, 31 cell types were identified in snRNA‐seq and 33 cell types in snATAC‐seq (Figure 4d). The expression levels of the selected marker genes across different cell types of this study were shown (Figure S23a, Supporting Information). For example, astrocytes, whose major cell populations specifically highly express Gfap, Aqp4, Slc4a4, Slc1a2, illustrating the accuracy of cell type definition in this study. These cell types can be broadly categorized into four major classes: Neurons, Oligodendrocytes (Oligos), Microglia and Immunocytes (Microglia_Immunocytes), and Astrocytes and Vascular Structure cells (Astrocytes_Vasc_Struct) (Figure 4e; Figure S22d, Supporting Information). The variations in cell type abundance within the dataset were also assessed (Figure S21e–h, Supporting Information). The overall changes in cell type proportions permit the visual observation of a reduction in glial cells following SCI in the snRNA dataset (Figure S21i, Supporting Information). However, no significant differences in cell type occupancy were found between conditions in the snATAC dataset (Figure S21j, Supporting Information). This discrepancy may be due to its technical nature, and snRNA‐seq measures RNA expression levels within each cell, making it more sensitive in detecting cell types and their changes. Furthermore, it is important to highlight that negatively correlated compositional count data may also obscure genuine variation between specific cell types. This is due to the inherent competition effect inherent in this data structure, whereby an increase in one cell type leads to a relative decrease in other types. Furthermore, it is necessary to highlight a potential ambiguity regarding the percentage of cell types. In both the snATAC and snRNA datasets of the present study, astrocytes exhibited a low percentage of ≈0.4% across all conditions (Figure S23b,c, Supporting Information). To illustrate this issue, we conducted a comparative analysis of the different methods.^[^ ^16^, ^22^ ^]^ We compared the following factors: tissue type, storage methods, whether cells or nuclei were used, single cell sequencing methods, lysis buffers, isolation methods, and density gradient centrifugation protocols (Figure S23d, Supporting Information). We also demonstrated the proportions of cell types in each study, highlighting discrepancies in the efficiency of cell type capture. By comparing the different methods in the table, potential explanations for the low percentage of astrocytes in this study can be discerned. It is important to note that the use of nuclei for single‐cell sequencing may result in the loss of activation‐related genes (e.g., APOE, CST3, FTL, SPP1, B2 M, PLD3, and CD74).^[^ ^22^ ^]^ This phenomenon was similarly observed in our dataset, where, for example, genes associated with the activation of microglia (e.g., Ccl4, Ccl3, Spp1, Lpl1) were largely undetected (Figure S23e,f, Supporting Information). In any case, these findings will provide some guidance for subsequent research projects or researchers in the same field with regard to experimental design and optimization.

In order to gain a more precise insight into the impact of varying conditions on cell type occupancy, we used Bayesian modeling to evaluate changes in the subpopulations after injury or therapy (Figure 4f). Compared with the sham group, myelinating oligodendrocytes (MFOLs) were significantly reduced after SCI, possibly due to secondary injury, leading to the reduction in sustained oligodendrocytes death and axonal demyelination.^[^ ^23^ ^]^ Compared with the injured group, MFOLs were significantly increased in the therapy group. Moreover, Ce@UCNP‐BCH may have accelerated axonal remyelination. This inference is corroborated by the results of differential gene expression between conditions in major cell types (Figure 4g). Following SCI, there is a reduction in expression levels of genes (e.g., Plp1 and Cnp) associated with myelination, while following treatment with Ce@UCNP‐BCH, there is an increase in the expression of these genes. A similar situation was observed with genes (e.g., Adcyap1r1 and Bdnf) associated with the negative regulation of ROS signaling pathway as well as the nerve growth factor signaling pathway. In addition to our previous in vivo and in vitro histological analyses, we further demonstrated that Ce@UCNP‐BCH treatment can effectively promote neuronal regeneration and myelin formation.

For activated_microglia, the proportion of cells significantly increased after SCI and after Ce@UCNP‐BCH treatment. However, the effects of activated microglia after SCI should be taken into consideration. For example, activated microglia can release pro‐inflammatory factors that worsen the secondary injury. Activated microglia can also release anti‐inflammatory factors, inhibit inflammatory responses, and clear the debris.^[^ ^24^ ^]^ Meanwhile, microglia depletion can worsen inflammation after SCI, inhibit functional recovery, and reduce macrophage infiltration.^[^ ^25^ ^]^ Therefore, the mechanism of action of activated microglia requires further investigation. It is noteworthy that Ce@UCNP‐BCH significantly increased leptomeninges compared with the injured group (Figure 4f). Furthermore, we observed an increase in the expression of genes (e.g., Ocln and Ctnnb) related to the tight junction assembly signaling pathway after treatment (Figure 4g). These finding indicating the re‐establishment of the BSCB after Ce@UCNP‐BCH treatment.

### 2.7 Ce@UCNP‐BCH Promotes Neuronal Regeneration and Myelin Formation

**Figure 5:**
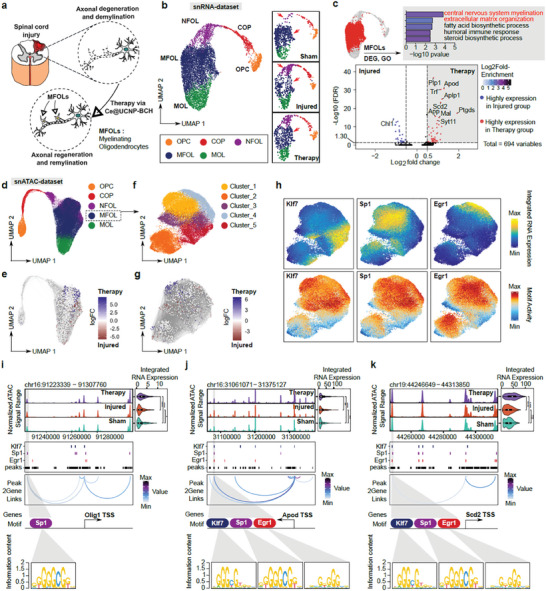
Gene regulatory dynamics of remyelination after treatment. a) Schematic of remyelination after Ce@UCNP‐BCH treatment following SCI. b) UMAP plot showed cell types of oligodendrocytes in snRNA‐seq, the clusters of cell types under three conditions: Sham, Injured, and Therapy were shown on the right, red arrow highlighted differences in cell populations. c) Enhanced Volcano plot showed differential gene expression in I (Injured) and C (Therapy) in MFOLs in snRNA‐seq. Positive values indicated high expression in condition C. The enrichment of the GO terms for the highly expressed genes was shown in the inset bar chart. d) UMAP plot showed cell types of oligodendrocytes in snATAC‐seq, annotation by using label transfer from snRNA‐dataset. e) Differential abundance of oligodendrocytes between condition I (injured) and condition C (Therapy) using MiloR. Colored spots represent neighborhoods that are differentially abundant with spatial (FDR < 0.1); Positive values indicated that the cell types were enrich in the condition C (Therapy). f) UMAP plot showed sub clusters of MFOLs in snATAC‐seq, computed the iterative LSI dimensionality reduction with Peak Matrix. g) Same as e, but for MFOLs between condition I (injured) and condition C (Therapy), positive values indicated that the cell types were enrich in the condition C (Therapy). h) Selected marker gene expression and TF motif activity deviation z‐scores for sub clusters of MFOLs. i) Genomic tracing of Olig1 accessibility for different conditions. The integrated RNA expression levels were shown in a violin plot to the right of each condition. Selected transcription factors were highlighted in the feature tracks using different color. Loops below the tracks indicate peak‐to‐gene connections. TFs Motifs that potentially regulate Oligo1 gene expression are shown at the bottom. j) Same as i, but for Apod gene. k) Same as i, but for Scd2 gene.

In models of spinal cord contusion, the dorsal neuron is typically the area of initial direct mechanical damage. Interestingly, the number of dorsal neurons significantly decreased after SCI and significantly increased after Ce@UCNP‐BCH treatment (Figure S24e, Supporting Information). Neuron types and location information were defined as described by Russ et al.^[^ ^16c^ ^]^ (Figure S24a–c, Supporting Information). Furthermore, dorsal neuron types were more separated, while ventral neuron groups were overlayered (Figure S24d, Supporting Information), like the conclusions of Yadav et al.^[^ ^26^ ^]^ In light of the aforementioned evidence, we postulate that Ce@UCNP‐BCH may promote neuronal regeneration and myelin formation (**Figure** **5**a). The oligodendrocyte subpopulations were grouped into subsets for quality control and clustering (Figure S24f,g, Supporting Information). A visual examination of the data reveals that the number of cells in NFOLs is increasing in comparison to the Sham group, while the number of cells in MFOLs is decreasing. Following the treatment, the number of cells in MFOLs increases (Figure 5b). It is possible that the results of overall analyses may conceal minor yet significant biological alterations. Consequently, we proceeded to subset the MFOLs in order to facilitate a comparison of their respective gene expression profiles under varying conditions (Figure 5c). The Ce@UCNP‐BCH treatment resulted in a significant increase in the expression of Plp1, Apod, Trf, Aplp1, and Scd2 in comparison to the Injured group. The biological processes related to these genes included central nervous system myelination, extracellular matrix organization, fatty acid biosynthetic process, humoral immune response, and steroid biosynthetic process. The activation of these bioprocess pathways following treatment indicates the potential for positive biological effects of the treatment in terms of promoting nerve and tissue repair, enhancing metabolic support and immune regulation, and contributing to functional recovery from SCI. Regeneration and myelination of nerve axons and reconstruction of the cytoskeleton require energy. Oxidative phosphorylation in mitochondria provides ≈95% of the total ATP in higher animal cells.^[^ ^27^ ^]^ Gene set enrichment analysis (GSEA) showed that Ce@UCNP‐BCH induced activation of cardiac muscle contraction, oxidative phosphorylation, metabolic pathway, mineral absorption, and phagosome (Figure S24h, Supporting Information). These results suggest that Ce@UCNP‐BCH can improve bioenergetic metabolism and coordinate energy supply for restoring homeostasis and reducing inflammation.

We integrated the snATAC with the snRNA dataset to further investigate the gene regulatory mechanism of neuronal regeneration and myelin formation (Figure 5d; Figure S25a, Supporting Information). Results demonstrated specific chromatin accessibility in different cell types and was highly correlated with gene expression (Figure S25b, Supporting Information). Clusters 5, corresponding to MFOLs, strongly enriched SOX family, SP family, and Klf7 (Figure S25c, Supporting Information). The overexpression of Sox11 and Klf7 has been demonstrated to facilitate axon regeneration.^[^ ^28^ ^]^ To more intuitively resolve the heterogeneity of cellular components, we used Milo to perform differential abundance analysis of cellular components between conditions. In contrast to the injured group, the Ce@UCNP‐BCH group exhibited a greater enrichment of MFOLs (Figure 5e; Figure S25d, Supporting Information). This finding is in accordance with those previously observed using scCODA. To resolve the heterogeneity of MFOLs and explore the changes of MFOLs perturbation by drug treatment, we subset MFOLs in the scATAC dataset to perform dimensionality reduction again and identified five sub‐clusters (Figure 5f). We also performed differential abundance analysis of cellular components between conditions in these five sub‐clusters using Milo. In contrast to the injured group, the Ce@UCNP‐BCH group exhibited a greater enrichment of Cluster_1 (Figure 5g; Figure S25e, Supporting Information). Cluster_1 displayed high gene expression levels associated with energy production, myelination, and cell growth functions, including Glul, Srebf1, Srebf2, Cnp, Ogdhl, Tgfb3 and Nr4a1 (Figure S25g, Supporting Information), as well as a high enrichment for transcription factors that regulate neuronal development, repair, and stress response, such as Klf7, Sp6, Sp1, Klf4, Egr1 (Figure 5h; Figure S25f, Supporting Information). Biological process of Gene Ontology (GO) annotations related to these genes include structural constituent of myelin sheath, L−serine transmembrane transporter activity, and negative regulation of respiratory burst (Figure S25h, Supporting Information). The activation of these biological pathways indicates that the treatment may have a positive effect on the formation of myelin and the regulation of oxidative stress, which in turn may contribute to the functioning of cells and the repair of tissues.

To investigate the gene regulatory dynamics of remyelination following treatment, we utilized snATAC‐seq data to compare the chromatin accessibility profiles across different conditions (Figure 5i,j). In this specific region, we identified peaks in chromatin accessibility that correspond to regulatory elements potentially involved in remyelination. The presence of Klf7, Sp1, and Egr1 binding sites within these peaks suggests that these transcription factors may play crucial roles in the gene regulation dynamics of remyelination. The Peak2Gene links further illustrate the connection between these regulatory elements and the target gene Olig1, Apod, and Scd2, which is critical for oligodendrocyte differentiation and myelination. For example, the motif analysis reveals the Sp1 binding motif within these regulatory elements, supporting the hypothesis that Sp1 may regulate Olig1 expression during remyelination. It is noteworthy that although integrated RNA expression data indicates significant differences among these groups, with higher expression levels observed in the therapy group compared to the injured and sham groups, there was no significant difference in chromatin accessibility between the different groups. This indicates that treatment may enhance Olig1 gene expression through post‐transcriptional or transcriptional regulatory mechanisms, such as the activation of transcription factors, rather than by altering chromatin accessibility. Overall, this integrative approach, which combines chromatin accessibility data with gene expression analysis, highlights the complex regulatory networks underlying remyelination and underscores the potential therapeutic effects of Ce@UCNP‐BCH in promoting spinal cord repair by modulating these gene regulatory dynamics.

### 2.8 Mechanism Underlying the Therapeutic Effect of Ce@UCNP‐BCH

**Figure 6:**
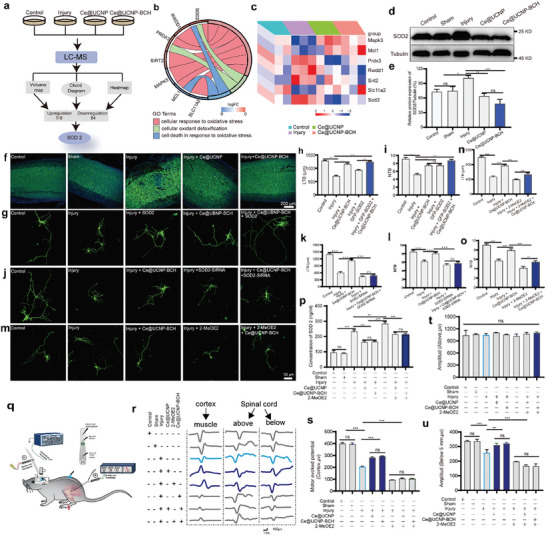
Mechanism involved in the neural restoration behaviors of Ce@UCNP‐BCH in vitro. a) The experimental framework of liquid chromatography‐mass spectrometry (LC‐MS) showing the target proteins of Ce@UCNP‐BCH associated with inflammation. b) Chord diagram showing the differentially expressed proteins of Ce@UCNP‐BCH targets involved in three pathways: cellular response to increased oxygen level, cellular oxidant detoxification, and cell death in response to oxidative stress. Individual connecting curves indicate distinct protein functions within cells. c) Heatmap showing the protein expressions in different groups for (b). d) Western blot analysis showing SOD2 expression levels in mouse spinal cord tissue in the control, sham, injury, injury + Ce@UCNP, and injury+ Ce@UCNP‐BCH groups. e) Densitometric quantifications of western blots depicted in (d). f) Immunofluorescence fluorescent images of spinal cord tissue stained with anti‐SOD2 antibodies, scale bar = 200 µm. g) Confocal microscopy fluorescence images of neuron in control, injury, injury + SOD2, injury + Ce@UCNP‐BCH, and injury + Ce@UCNP‐BCH + SOD2 groups, scale bar = 50 µm. h,i) LTB and NTB of the total number of branches for (g). j) Confocal microscopy fluorescence images of neuron in control, injury, injury + SOD2‐SiRNA, injury + Ce@UCNP‐BCH, and injury + Ce@UCNP‐BCH + SOD2‐SiRNA groups, scale bar = 50 µm. Measured j's total branch LTB (k) and NTB (l) in terms of relative length. m) Confocal microscopy fluorescence images of neurons in control, injury, injury + Ce@UCNP‐BCH, injury + 2‐MeOE2, and injury + Ce@UCNP‐BCH + 2‐MeOE2 groups, scale bar = 50 µm. Quantified LTB (n) and number NTB (o) of total branches for (m). p) ELISA analysis showing SOD2 levels in serum of mouse in control, sham, injury, injury + Ce@UCNP, injury + Ce@UCNP‐BCH, injury + 2‐MeOE2, injury + Ce@UCNP + 2‐MeOE2, injury + Ce@UCNP‐BCH + 2‐MeOE2 groups. q) The schematic diagram of the electrophysiological experiment. r) Representative images during the electrophysiological measurements. s, t, and u. Cortical motor evokes potentials on the left side, and amplitudes of electrical signals in the spinal cord above and below the injured segments in each group (n = 3). * means *p* < 0.05, ** means *p* < 0.01, *** means *p* < 0.001, ns means not significant.

The results demonstrated that Ce@UCNP‐BCH improves hindlimb motor function recovery in SCI mice and promotes the regeneration of injured spinal cord tissue by enhancing neuronal survival, inhibiting glial scar formation, and recruiting eNSPCs. However, the underlying molecular mechanisms were further assessed via proteomics and bioinformatics analysis. The experimental design of LC‐MS for identifying target proteins of Ce@UCNP‐BCH is shown in **Figure** **6**a. A total of 202 differentially expressed proteins were detected between the injury and Ce@UCNP‐BCH groups (118 upregulated and 84 downregulated). These proteins were visualized on a volcano map (Figure S26a, Supporting Information). Further analysis revealed that the differentially expressed proteins involved in cellular responses to increased oxygen levels, cellular oxidant detoxification, and cell death under oxidative stress were illustrated in a chord diagram. SOD2 was the only protein identified as participating in all three functional pathways (Figure 6b). Heatmap analysis showed that SOD2 expression was significantly higher in the SCI group than in the Ce@UCNP‐BCH group (Figure 6c). Western blot analysis confirmed this, showing elevated SOD2 levels in both cortical neurons and spinal cord tissue in the SCI group, with decreased expression following Ce@UCNP‐BCH treatment (Figure 6d,e; Figure S26b,c, Supporting Information). SOD2 antibody immunofluorescence staining further supported these findings (Figure 6f). The Ce@UCNP‐BCH acts as a metalloenzyme, directly enhancing SOD2's enzymatic activity, thereby increasing its capacity to detoxify ROS.^[^ ^29^ ^]^ Unlike the untreated injury group, which shows compensatory upregulation of SOD2 due to elevated oxidative stress, Ce@UCNP‐BCH effectively reduces ROS by augmenting SOD2 activity.^[^ ^30^ ^]^ This catalytic enhancement allows ROS clearance without the need for increased SOD2 expression.

To further validate the synergistic effect of Ce@UCNP‐BCH and SOD2, the GFP‐SOD2 plasmid was successfully transfected into hippocampal neurons, where it was observed that neuronal repair significantly improved following transfection, and this effect was further enhanced with Ce@UCNP‐BCH treatment (Figure 6g–i). Conversely, the use of SOD2‐SiRNA to silence SOD2 expression inhibited the therapeutic effects of Ce@UCNP‐BCH (Figure 6j–l; Figure S27c–f, Supporting Information), confirming the role of SOD2 in the drug's mechanism. The above used SOD2‐SiRNA2 for the highest SOD2 interference rate, which was verified by Western blot (Figure S27a,b, Supporting Information).

Furthermore, SOD2 inhibitor, 2‐MeOE2,^[^ ^31^ ^]^ was used to explore the mechanism of the therapeutic effect of Ce@UCNP‐BCH in vitro and in vivo. Adding 2‐MeOE2 to hippocampal neurons negated the recovery effects of Ce@UCNP‐BCH (Figure 6m–o). Electrophysiological assessments of motor evoked potentials (MEPs) and spinal cord electrical conduction post‐injury showed that Ce@UCNP and Ce@UCNP‐BCH treatments significantly increased MEP amplitudes, reflecting improved corticospinal tract function. However, 2‐MeOE2 treatment significantly diminished these effects (Figure 6q–u). Finally, ELISA assays were used to measure SOD2, IL‐6, and TNF‐α levels in serum samples from mice (Figure 6p; Figure S28, Supporting Information). SOD2, IL‐6, and TNF‐α concentrations were notably higher in the injury group compared to control and sham groups. Treatment with Ce@UCNP or Ce@UCNP‐BCH reduced these levels, suggesting a decrease in oxidative stress and inflammatory response. The addition of 2‐MeOE2 reversed these effects, confirming that Ce@UCNP‐BCH enhances the functional recovery of SCI mice primarily by regulating SOD2 activity to manage oxidative stress and inflammation. These findings illustrate that Ce@UCNP‐BCH facilitates functional recovery by enhancing SOD2 activity, thus providing effective ROS detoxification without necessitating the compensatory overproduction of SOD2.

## 3 Discussion

Traumatic SCI triggers a complex temporal cascade of events, ultimately leading to the formation of a complex network of multiple cell types, including extracellular components and non‐neural components, thus preventing spontaneous axonal regeneration.^[^ ^32^ ^]^ In this study, an advanced nanoprobe loaded with CeO_2_ NPs that could effectively eliminate ROS and simultaneously monitor the damage repair in real‐time using a ratiometric UCL signal was developed. The probe could also improve the realization of the ETLSA strategy for optimal therapeutic efficiency. Meanwhile, results showed that axonal remyelination can be identified using snATAC‐seq and snRNA‐seq by observing the dynamic changes in each cell type within the spinal cord following injury and treatment. Furthermore, the ETLSA strategy could synergistically boost ROS consumption through the SOD‐related pathways after SOD‐siRNA treatment.

In this study, we applied an advanced Ce‐based nanozymes for SCI treatment which has strong anti‐oxidative stress properties and can protect neurons. In addition, these synthetic nanoparticles can traverse the BSCB, facilitating controlled drug release and the precise delivery in terms of timing, location, and dosage of drugs or small active molecules while maintaining effective concentrations. Several studies have demonstrated the high efficacy of Ce nanozymes in scavenging ROS and protecting neuronal functionality.^[^ ^6^, ^7^, ^32^ ^]^ Our research results also confirm the effectiveness of these methods in promoting the regeneration of spinal cord tissue, including the myelin sheath, and additionally improving motor function in mice suffering from spinal cord injuries. Although preclinical studies have demonstrated that these materials have shown good therapeutic effects by alleviating oxidative stress, they cannot simultaneously monitor the extent of oxidative stress or injury at the site of SCI, which is an important aspect in the evaluating of therapeutic effects.^[^ ^33^ ^]^ However, it is currently challenging to develop an approach for monitoring the exact level of oxidative stress in situ of SCI.^[^ ^34^ ^]^ Previously, various optical probes including fluorescent sensor were found to effectively monitor the changes in redox state,^[^ ^35^ ^]^ but these probes have poor resolution and may be susceptible to interference by various factors including the limited tissue penetration.^[^ ^36^ ^]^ This makes them unable to reflect the actual redox state. According to the ETLSA strategy, we doped CeO_2_ NPs with upconversion materials and loaded it with a coumarin derivative dye which possesses a H_2_O_2_‐responsive double bond (BCH), serving a dual purpose as a highly responsive indicator of oxidative stress. Furthermore, our data showed that the ratiometric UCL signal (650/690 nm) can be utilized for real‐time monitoring of the dynamic changes in the redox state in spinal cord injuries. However, the optimal delivery mode and long‐term adverse effects were not determined. Moreover, further optimization of the nanoparticle structure and monomer ratio is required to enhance diffusion and improve delivery efficiency. Additionally, although nanozymes exhibit individual efficacy in addressing oxidative stress and offering neuroprotection, it is imperative for future research to explore synergistic approaches that can enhance the mitigation or monitoring of oxidative stress. It's worth emphasizing that these limitations are still relevant within the framework of our present study.

In this study, single‐cell sequencing was performed to investigate and reveal the intrinsic mechanisms through which nanoenzymes promote SCI repair. By comparing changes in the abundance of cellular components and gene expression between different conditions, results indicated that Ce@UCNP‐BCH treatment after SCI enhanced neuronal regeneration and coordinated oligodendrocytes thereby accelerating neuronal axon remyelination, which promotes signaling and tissue remodeling as well as the recovery of mitochondrial function after SCI. It also activates the signaling pathways involved in the regulation of energy supply to support the key processes of regeneration, such as cytoskeletal reconstruction, nutrient transport, and axon remodeling. Interestingly, the LFB/TUNEL staining and immunostaining analysis provide a comprehensive assessment of the relative integrated morphology, encompassing myelin structures. This suggests that nanoenzymes have the potential to reduce demyelination and thereby protect nerve fibers. Besides, results of the NeuN/Brdu co‐staining and NFH/GFAP co‐staining showed a similar trend in promoting neuron regeneration and glial scar formation. A recent study involving a multimodal single‐cell and spatial atlases of SCI, comprising over 500000 cells aimed to bridge the fields of epigenomics and transcriptomics to elucidate gene expression regulatory networks while addressing the complexities as function of spatial and temporal heterogeneity.^[^ ^37^ ^]^ In addition, previous studies have effectively created a cell atlas that delineates distinct regions within the injured rhesus monkey spinal cord across different phases from acute to chronic. This atlas effectively depicts the cellular heterogeneity and space‐time dynamics of different cell types in the injured rhesus monkey spinal cord.^[^ ^38^ ^]^ Elsewhere, a comprehensive single‐cell atlas of SCI in mice has recently been reported, which identified a specific pro‐regenerative signature in spinocerebellar neurons.^[^ ^16^ ^]^ This highlights the growing incorporation of single‐cell multi‐omics approaches to investigate specific disease models and identify potential therapeutic interventions. However, the aim of our study was to elucidate the cellular and molecular landscape of the injured or treated spinal cord using Ce@UCNP‐BCH through an innovative exploration into its application for SCI. Nonetheless, there were several limitations in single‐cell research in this study. We primarily focus on the cell nucleus, which means that mRNA information in the cytoplasm is not captured. However, this approach can avoid selective cell death and experimentally induced gene expression while stimulating the capture of larger cell types that may have otherwise be undetected due to their size. Currently, droplet or microwell‐based single‐cell sequencing technologies show an average reaction chamber size in the range of 30–100 microns, which effectively filters these larger cells. Moreover, by focusing on the homogeneous nucleus, this issue is resolved. Although there may be differences in mRNA information between the nucleus and whole cells, the abundant transcriptional data within the nucleus allows for the accurate identification of specific cell types.^[^ ^39^ ^]^ Furthermore, the present study only collected omics data at a single time point after SCI, without observing temporal changes in diverse cell types. Nevertheless, the main aim of this study was to investigate drug‐induced mechanisms involved in the repair of SCI under diverse conditions. This article presents a comprehensive overview of all cell types applied in pre‐ and post‐SCI treatment, with emphasis on the molecular mechanisms associated with drug‐induced alterations in oligodendrocytes which drive molecular‐level spinal cord repair. Currently, few studies have focused on neurons and small glial cells. In future studies, it is crucial to reconstruct spatiotemporal molecular landscapes during SCI by integrating spatial genomics techniques such as stereo‐seq^[^ ^40^ ^]^ with interconnecting different temporal nodes or transforming animal models into non‐human primate models that are anatomically similar to humans.

To provide a deeper understanding of the molecular mechanism behind the mentioned effects, we conducted thorough proteomic and bioinformatics analysis, leading to the successful identification of SOD2 as the target protein. SOD2, a member of the iron/manganese superoxide dismutase family, has a homotetrameric structure and can bind one manganese ion per subunit.^[^ ^41^ ^]^ It has been shown to catalyze the disproportionation of superoxide anion radicals to generate oxygen and hydrogen peroxide. This enzyme is involved in the maintenance of redox balance in the body and has been linked to both onset and progression of SCI.^[^ ^42^ ^]^ Our research findings illustrate that SOD2 was upregulated in the Ce@UCNP‐BCH group. Moreover, modulating the expression of SOD2 influenced the therapeutic efficacy of Ce@UCNP‐BCH, which was consistent with the findings from single‐cell sequencing analysis. These results suggest that SOD2 can mediate treatment effects. However, it is important to acknowledge that other proteins may also play a role in this phenomenon. Furthermore, the possibility of these proteins coexisting and synergistically interacting with SOD2 cannot be ignored. The precise molecular and signaling pathways that inhibit oxidative stress following SCI need to be clarified.

Overall, we report for the first‐time cerium‐doped upconversion nano enzymes that act as ratio metric luminescence probe for can real‐time evaluation of the severity of SCI and simultaneously decreasing excessive ROS, thereby guide the formulation of dosage and timing of drug administration, hence improve the personalized treatment of SCI.

## 4 Experimental Section

### Synthesis of Ce@UCNP

First, Y(CH_3_COO)_3_•4H_2_O (0.78 mmol), Yb(CH_3_COO)_3_•4H_2_O (0.20 mmol), Er(CH_3_COO)_3_•4H_2_O (0.015 mmol), and Ce(CH_3_COO)_3_•4H_2_O (0.005 mmol) were mixed with 6 mL oleic acid, and 15 mL 1‐octadecane in a flask. The solution was heated to 160 °C to form a homogeneous solution and then cooled down to room temperature. 10 mL methanol solution containing NaOH (2.5 mmol) and NH_4_F (4 mmol) was slowly added into the flask and stirred for 30 min. After that, the solution was heated and degassed at 120 °C for 30 min to remove methanol, then heated to 300 °C and maintained for 1.5 h under argon protection. When the solution was cooled naturally, nanoparticles were precipitated from the solution with ethanol and collected by centrifugation (6000 g, 5 min), and then washed with ethanol/cyclohexane (9:1, v/v) for three times. The precipitate as the Ce@UCNP could be redispersed in cyclohexane.

### Synthesis of Ce@UCNP‐BCH

In a typical procedure, DSPE‐PEG_2000_ (5 mg) was dissolved in 1 mL DI water, then the chloroform solution of oleic acid‐coated UCNPs (Ce@UCNP) (500 µL, 10 mg·mL^−1^) were added to the above solution, subjected to ultrasound for 2 min, and stirred overnight at room temperature. After chloroform evaporated, the rest material dispersed easily in water. After centrifugation twice, the products were washed with DI water and stored in DI water for further functionalization. BCH^[^ ^43^ ^]^ (5 mg) was dissolved in dimethyl sulphoxide (DMSO, 50 µL) and added dropwised into DI water (2 mL) containing polyethylene glycol (PEG) functionalized Ce@UCNP (20 mg). The solution was then stirred overnight. Free BCH was removed by centrifugation at 10000 rpm for 10 min. The precipitate was washed three times with phosphate buffer saline (PBS) The as‐obtained hybrid materials (Ce@UCNP‐BCH) were redispersed in PBS solution (2 mL, pH 7.4) and stored at 4 °C.

### Primary Hippocampal Neurons Culture

All operations were performed in accordance with procedures of the First Affiliated Hospital of Jinan University Institutional Animal Care and Use Committee. P0 (1‐day‐post‐birth) Sprague‐Dawley rats were acquired from Animal Experiment Center of Southern Medical University. The hippocampi were dissected from the pups and digested using 0.125% trypsin (Gibco; 25200‐072) for 25 minutes at 37 °C. Next, a mixture of DMEM/F12 medium and 10% fetal bovine serum was added to terminate the digestion process. Next, the mixture was allowed to stand until the tissue block settled, and the supernatant was carefully removed. Following this, an appropriate volume of culture medium was added, and a glass dropper was used to gently separate the neurons from the digested hippocampal tissue. Moreover, the cells were seeded on glass coverslips which were pre‐coated with poly‐D‐lysine (Gibco; P6407) at cell density being 1 × 10^4^ cells/cm^2^. The medium was changed to Neurobasal‐A medium (Gibco; 10888‐022) supplemented with 2% B27 (Gibco; 16954044) after 6 h of incubation. To ensure an optimal environment for neuron culture, half of the medium was replaced every three days. The duration of in vitro culturing was determined based on the specific goals of the experiments.

### Cell Viability Assay

Cell Counting Kit‐8 assay was used to evaluate the cytotoxicity. The HT‐22 cells or hippocampal neurons were seeded in 96‐well culture plates at a cellular density of 1 × 10^4^ cells/cm^2^, with three multiple holes in each group. After culture for 12 h, the cells were incubated in the corresponding medium containing different concentrations of CeO_2_ NPs, Ce@UCNP, and Ce@UCNP‐BCH. After 12 h, the CCK‐8 detection reagent was prepared and added into the 96 well plates for 30 min. Next, the 96‐well plate reader from Thermo was utilized to record the absorbance values at a wavelength of 450nm. Finally, the cell viability was analyzed using GraphPad Prism 7.

### Detection of Cellular ROS

Cellular ROS was measured using 2′,7′‐Dichlorodihydrofluorescein diacetate (DCFH‐DA, S0033S, Beyotime) and dihydroethidium (DHE, S0063, Beyotime). The HT‐22 cells were cultured on cell crawls in 24‐well plates and then cultured in a medium containing glutamate for 12 h. The medium was replaced with a medium containing the different concentrations of CeO_2_ NPs, and Ce@UCNP. After 12 h, the DCFH‐DA or DHE detection reagents were added into the 24‐well plates for 30 min. The cells were then washed with phosphate buffer saline (PBS) to remove the residue culture medium. Subsequently, precooled 4% paraformaldehyde was added into each well for 45 min at 4 °C in dark room. The 4% paraformaldehyde was removed and washed with PBS. The cell membrane was permeabilized through three consecutive 10‐min treatments with TBST containing 1% Triton X‐100. Subsequently, the cells were treated with a mounting solution that contained DAPI (EMS, Hatfield, PA). Finally, microscopic examination and image analysis were conducted using confocal microscopes (Carl Zeiss, Germany).

Moreover, ROS levels in cells were quantified using flow cytometry. HT‐22 cells were added into 6‐well culture plates. Next, the cells were incubated in the corresponding medium enriched with glutamate for 12 h. The medium was discarded, and the cells were cultured in a medium containing varying concentrations of CeO_2_ NPs and Ce@UCNP for a duration of 12 h. The cells were washed with PBS. Next, the cells were digested using trypsin from the culture bottle wall and collected into a 15 mL centrifuge tube. It was then centrifuged for 5 min at a speed of 5000 rpm/min. The supernatant was removed and 500 µL PBS was added into a 15 mL centrifuge tube to suspend the cells. Next, the contents of the cell were transferred into a centrifuge tube with a volume of 1.5 mL. First, the sample was centrifuged at 5000 rpm for 5 min. The supernatant was carefully aspirated, and then 300 µL of PBS containing DCFH‐DA detection reagents (v/v = 1:1000) was added and mixed. This mixture was left at room temperature for 20 min. Afterward, the sample was centrifuged again at 5000 rpm for 5 min, the supernatant was aspirated, and 300 µL of PBS was added and mixed. It was then left in a dark room for another 20 min. For fluorescence analysis, a 488 nm excitation beam from the FACS Array Bioanalyzer (BD Biosciences) was used. The mean green fluorescence intensity was quantified using Flow Jo 7.6 software.

### Immunocytochemistry

Neurons were cultured on glass coverslips pre‐coated with poly‐D‐lysine (PDL, Gibco, P640) in 24‐well culture plates. After specific treatments, the neurons were fixed at a temperature of 4 °C for 45 min using a precooled 4% PFA. Next, the PFA was removed, and the residue was washed with PBS. The cell membrane was permeabilized with the TBST solution containing 1% Triton X‐100, with three 10 min each. The neurons were blocked for an hour at room temperature using 3% albumin bovine serum in TBST with 1% Triton X‐100. The cells were incubated with an anti‐βIII Tubulin antibody of mouse origin (Abcam, ab18207) and/or an anti‐SOD2 antibody of rabbit origin (Abcam, ab68155) at 4 °C for 12 h. Subsequently, the cells were washed three times with TBST containing 1% Triton X‐100, with each wash lasting 10 min. The cells were cultured with anti‐Rabbit Alexa Fluor 647 (Abcam, ab150075) or anti‐Mouse Alexa Fluor 488 (Abcam, ab150073) for 2 h. The cells were washed three times and then treated with a mounting solution that contained DAPI (EMS, Hatfield, PA). Microscopy and image analysis were performed using confocal microscope (Carl Zeiss, Germany).

### Spinal Cord Bruise Model

The mouse spinal cord Bruise model was established as described previously.^[^ ^44^ ^]^ Briefly, mice were anesthetized using a 1.25% solution of tribromoethanol. (Sinopharm Chemical Reagent Co., Ltd., Beijing, China) through intraperitoneal injection of 400 µL/20 g. Next, a dorsal incision was made longitudinally to expose the laminae of T9–11, with a length of 2.0 cm. The T10 lamina was completely removed to expose the spinal cord tissue for better visibility. Next, a U‐shaped stabilizer (University of Louisville) was employed to secure the T10 facets in place. The stabilizer was adjusted to the appropriate position on the Louisville Injury System Apparatus platform. The precise location of the spinal cord impact was determined using a red laser beam. And the strike was set at a striking depth of 0.8 mm and a duration of 0.5 seconds at 18 psi (124 kPa). Following the injury, a microscopic examination was carried out to assess the affected area, followed by meticulous hemostasis. The muscles and skin were then sutured together. Mice that replicate SCI were selected for the study. These mice exhibited characteristic symptoms such as limp immobility, torso and leg twitching, reflexive tail wagging, as well as ischemia and edema around the injured spinal cord tissue.^[^ ^45^ ^]^ In addition, as for Sham‐operated animals, T10 total laminectomy was performed and no injury was caused on the spinal cord. Gentamicin was administered intramuscularly at a daily dosage of 2000 U to prevent infection. Every eight hours, the mice's bladders were manually compressed to facilitate urination until a natural voiding occurred. In the experimental group, specific materials were administered through intraperitoneal injection per 48 h for a period of five days, while in the injury group, all animals received a 200 µL saline solution via intraperitoneal injection for the same duration. The ethical approval from Jinan University was obtained prior to the research (approval number: 102641).

### Upconversion Imaging In Vivo

Multi‐mode animal live imaging system‐AniView 6100 （BLT） was used to detect Ce@UCNP‐BCH or Ce@UCNP up‐conversion imaging effect in vivo. After Ce@UCNP‐BCH was injected intraperitoneally, the SCI mice were anesthetized and placed on the imaging platform to up‐conversion image. The excitation and emission wavelengths were initially set at 980 nm and 650 nm, respectively, with detection of fluorescence intensity at 650 nm. Subsequently, the excitation and emission wavelengths were adjusted to 660 and 690 nm, respectively, for detection of fluorescence intensity at 690 nm. Finally, the ratio of fluorescence intensity at 690 nm to that at 650 nm was calculated.

### Detection of ROS In Vivo

Luminescent imaging system for small animals in vivo (Bidu imaging system, Ms Lumina I. Perkinelmei. USAnce) was used to measure the ROS level in vivo. The SCI mice were anesthetized, L‐012 (10 mg kg^−1^), a probe detecting the ROS level in vivo, was intraperitoneally injected. The anesthetized mice were put on the imaging platform for imaging. The fluorescence intensity indicated the level of ROS in vivo.

### Detection of the Inflammatory Factors In Vivo

Briefly, spinal cord bruise model was established and mice received the corresponding treatments. Three days later, blood was collected from the eyeball and centrifuged at 4 °C, 2000 g. The resulting blood serum was collected for the detection of TNF‐α, IL‐6, IL‐1β, and IL‐10 levels through ELISA kits.

### Behavior Test

The recovery of movement abilities of model mice was estimated through the open field test (OFT), Basso mouse scale (BMS) score and footprint test. The protocol of OFT has been described previously.^[^ ^46^ ^]^ Briefly, the mice were placed inside the corner container with dimensions of 50 cm × 50 cm × 40 cm. Each mouse was given 5 min to freely explore the arena to acclimate to the experimental environment. Then, they were allowed to move freely for 5 min within the corner box while a video camera positioned above captured their movements. The data were subsequently analyzed using the View Point system (Viewpoint SA, France). Following each test, the arena was cleaned with 75% alcohol. Within the 5‐min period, measurements were taken for the overall distance traveled, as well as the highest and average velocity. The BMS score was also used to examine the recovery well the SCI mice's hind limb locomotor function had recovered. Every three days following the injury, two blind experienced investigators conducted the analysis for five min. The evaluation criteria were described in detail previously.^[^ ^47^ ^]^

Moreover, the hind footprint test was performed to examine the motor performance of hindlimbs. Blue nontoxic paints were utilized to dye the hind feet. Subsequently, a runway with dimensions of 20 cm in length, 15 cm in width, and 15 cm in height was prepared for the mice. A sheet of white paper was placed on the floor, and the blue footprints of the mice were recorded on it. Stride measurements were collected for both width and length.

### H&E Staining and LFB Staining

H&E staining and LFB staining were performed to evaluate the changes in morphological structure and myelin sheath in the injured spinal cord tissue. Initially, 1.25% tribromoethanol was intraperitoneally injected to anesthetized mice at a dose of 20 µL g^−1^. The thoracotomy surgery was performed in a fume hood. After thoroughly exposing the heart, a blood vessel puncture needle was inserted into the left ventricle, while the right atrial appendage was incised. Following this, a solution of 0.9% NaCl was injected into the left ventricle along with the puncture needle to expel all blood. Next, a 4% PFA solution was introduced into the left ventricle to induce tissue hardening, thereby facilitating its complete extraction. The length of 1.2 cm of spinal cord tissue centered on the injury was collected. Next, the harvested tissues were embedded in paraffin which were cut into tissue sections of 10 mm thickness for tissue staining. Next, the sections were stained with hematoxylin and eosin (H&E). The procedures of H&E staining have been detailed elsewhere.^[^ ^48^ ^]^ Luxol fast blue (LFB) staining was performed on spinal cord slices to assess the extent and severity of demyelination resulting from contusion injury. The procedures of LFB staining were reported previously.^[^ ^49^ ^]^ A light microscope was used to scan and analyze the two stained sections.

### Immunofluorescence Staining

Initially, 50 mg Kg^−1^ BrdU was intraperitoneally injected into mice by one injection 5 days before execution, respectively.^[^ ^50^ ^]^ After embedding and sectioning the harvested spinal cord tissue as described above, the tissue on the slices was permeabilized using TBS‐Triton X‐100 for three rounds, each lasting 10 min. Following this, the tissue was blocked with a 3% bovine serum albumin solution for 1 h at room temperature. After three washes with TBS‐Triton X‐100, the tissues were incubated overnight at 4 °C with the respective primary antibodies. Nuclei were stained with DAPI dye (0.25 mg mL^−1^). The study also utilized anti‐NeuN/anti‐Brdu (NeuN: 1:600, Abcam, ab236870; Brdu: 1:500, Abcam, ab152095) primary antibodies to assess the viability of neurons and anti‐NFH/anti‐GFAP (NFH:1:500, Servicebio, GB12143; GFAP: 1:500, Abcam, ab7260) primary antibodies to detect the ratio of neuron and astrocytes. The Tunnel staining (Abcam, ab206286) was performed to assess apoptosis at the site of injury. Moreover, anti‐SOD 2 (1:1000, Abcam, ab68155) was also utilized to quantify the expression of SOD 2 in injured spinal cord tissue. The slices were washed three times with TBST at room temperature. Subsequently, they were exposed to secondary antibody (1:500) for 2 h at room temperature. Next, the sections were washed with TBST two times, 10 min in each wash.

### Sample Preparation for Proteomics Based on Mass Spectrometry

Cortical neurons were isolated from one‐day‐old Sprague‐Dawley offspring. First, cortical tissues were acquired from the offspring, digested with trypsin with a concentration of 0.125% for 25 min at 37 °C. Next, an appropriate volume of DMEM/F12 medium containing 10% fetal bovine serum was added to terminate digestion. The tissue was allowed to stand and precipitated. The supernatant was discarded, and an appropriate volume of DMEM/F12 medium containing 10% fetal bovine serum was added. A pasteur pipette was used to delicately mix the tissue. After allowing it to settle and discarding the supernatant, this process was repeated. Next, the appropriate volume of NB‐27 medium was added. Cortical neurons were then separated from the tissue using a glass dropper, creating a suspension of cortical neurons. Finally, the cortical neuron suspension was filtered through a 0.45 µm pore diameter filter tip to remove any remaining tissue fragments. Next, the filtrate from the cells was placed onto a 10 cm dish that had been coated with PDL for 12 h. After 36 h, the cell plates were divided into three groups: control group, injury group, and L‐Glutamic acid (120 M) was added to replace the original medium in the injury group. L‐Glutamic acid (120 m) was added to replace the original medium in the injury group. In the Ce@UCNP‐BCH group, Ce@UCNP‐BCH and Glutamate were introduced into the medium. After a 12‐h incubation period, the cells were disrupted, and the complete set of expressed proteins was collected and stored at −80 °C.

### Data Analysis and Liquid Chromatography‐Mass Spectrometry

Frozen proteins were retrieved from the −80 °C refrigerator and placed on an ice surface. Subsequently, they were digested with trypsin. The acquired peptides were analyzed through nanoflow reversed‐phase liquid chromatography‐tandem MS under similar conditions as those of the high‐performance liquid chromatography. The Ultimate 5600 system (AB SCIEX, Framingham, CA, USA) equipped with a linear ion trap was utilized in the data‐dependent acquisition mode. This enabled to generate a comprehensive list of proteins exhibiting different levels of expression, encompassing both upregulated and downregulated proteins in the injury group as compared to the experimental group. Subsequently, the study visualized these differentially expressed proteins through the use of volcano plots. The differentially expressed proteins involved in cellular oxidative detoxification, cell death in response to oxidative stress, and cell response to oxidative stress were investigated using a chord diagram. Finally, a heatmap was utilized to display the expression of these proteins in different group.

### Plasmid Constructs, RNA Interference and Transfection

The cDNA of Rus norvegicus SOD 2 sequence (NM_017051.2) was chosen and subsequently inserted into a pEGFP‐C1 tag (Clontech, PaloAlto, CA, USA) through cloning. All the produced plasmids had their DNA sequenced before being converted to plasmid. RiboBio (Guangzhou, China) synthesized the SOD 2 siRNA and negative control siRNA (siRNA NC) fragments. The sequences of the primers are shown below:

SOD 2 siRNA 1F: CGCACAUUAACGCGCAGAUCATT
SOD 2 siRNA 1R: UGAUCUGCGCGUUAAUGUGCGTT
SOD 2 siRNA 2F: UACAGAUUGCCGCCUGCUCUATT
SOD 2 siRNA 2R: UAGAGCAGGCGGCAAUCUGU ATT
SOD 2 siRNA 3F: ACUAUCUGAAAGCCAUUUGGATT
SOD 2 siRNA 3R: UCCAAAUGGCUUUCAGA UAGUTT

All constructs underwent sequencing verification. Additionally, western blotting was employed to confirm the interference effect of SOD 2 siRNA 1–3. Using a previously described calcium phosphate technique, hippocampus neurons were transfected with plasmid or siRNA.^[^ ^51^ ^]^

### Western Blotting

Protein samples were extracted from the cells. Briefly, treated cortical neurons were lysed using a cell lysis buffer containing PMSF (working concentration is 1 mM) for 30 min on ice. Next, loosely attached cells were removed from the dish using a cell scraper made of cold plastic. Next, the cell suspension was carefully transferred into a 1.5 mL EP tube, making sure the tube was placed on ice. In the case of samples from spinal cord tissue, the study used liquid nitrogen for freeze‐drying. As the liquid nitrogen approached evaporation, the tissue was promptly ground with a pestle. The tissue lysis, containing a protease inhibitor, was added for a 30‐min lysis at low temperature. Throughout the process, it was made sure that the sample lysate did not come into contact with the bottom or walls of the tube. Ultrasound was performed at 10% power (650 W) for 5 seconds, followed by a 5‐second pause. This cycle was repeated 10–25 times, being careful to prevent the formation of bubbles. Subsequently, the sample was centrifuged at 12000 rpm for 15 min. The resulting supernatant was then collected and transferred into a fresh 1.5 mL EP tube. Subsequently, an appropriate amount of 5 × protein loading buffer was added to the sample and boiled at 95 °C for 5 min. Next, the protein samples underwent separation using SDS‐PAGE gel at a voltage of 120 V. Subsequently, the proteins were electrophoretically transferred from the SDS‐PAGE gel onto a polyvinylidene difluoride (PVDF) membrane for a duration of 60 min, with a pore size of 0.22 µm. The PVDF membranes were first blocked using a TBS‐Tween‐20 solution containing 5% fat‐free milk for an hour. Subsequently, they were incubated overnight at a low temperature with anti‐βIII Tubulin antibody from mouse (Abcam, ab18207) and anti‐SOD2 (Abcam, ab68155). The next day, the membranes were exposed to either anti‐rabbit or anti‐mouse secondary antibodies at room temperature for one hour. Membrane visualization was carried out using both the Odyssey Infrared Imaging System (Licor Biosciences, USA) and the ECL Imaging System (Clinx Science Instruments Co., Ltd., China).

### Statistical Analysis

Data were analyzed and presented using GraphPad Prism (Version 8.3.0), and SPSS software (Version 24.0). To assess the normal distribution of continuous variables, the Shapiro‐Wilk test and Q‐Q plots were utilized. The homogeneity of variance was tested. Continuous data were presented as mean ± standard deviation (SD). For normally distributed and with homogeneous variances, one‐way ANOVA and LSD post‐hoc tests were employed. Statistical significance was set at p < 0.05.

### Single Nucleus ATAC/RNA Sequencing

Briefly, spinal cord bruise model was established and mice received the corresponding treatments. After 10 days, the mice were euthanized. The skin and subcutaneous tissue were incised along the surgical scar to fully expose the SCI site. The muscles around the two segments above and below the injury were freed, centering on the injured segment. The vertebral plate was then opened by grinding off the vertebral arches on both sides to get the spinal cord tissues and immediately stored in liquid nitrogen until the nuclei were isolated. Next, the samples were cut and transferred to a Dounce homogenizer (2 mL Dounce Tissue Grinder Set, Sigma) containing a 2 mL lysis buffer (0.25 m sucrose, 20 mm Tris‐HCl [pH 7.8], 25 mm KCl, 5 mm MgCl2, 1 mM DTT, 1x protease inhibitor cocktail, 0.4 U/µL RNase inhibitor, 1% BSA). It was incubated for 2 min on ice and then gently homogenized using 10 strokes of pestle A, followed by 5–10 strokes of pestle B, and finally filtered through a 30 µm cell sieve into a 15 mL centrifuge tube. The filtered lysate was centrifuged at 500 × g for 5 min at 4 °C. Subsequently, the pellet was resuspended in 2 mL of nuclei wash buffer (lysis buffer: Iodixanol Solution = 3: 2) and Iodixanol Solution (20 mm Tris‐HCl [pH 7.8], 25 mm KCl, 5 mm MgCl2, 50% Optiprep). The tube was centrifuged at 800 × g for 10 min at 4 °C and the resulting pellet was resuspended in 0.1 mL of nuclei resuspension buffer (1% BSA in PBS, 0.4 U/µL RNase inhibitor). The efficiency of nucleus lysis was assessed using Taipan Blue, and the quality and count of nuclei were examined using DAPI staining.

Single‐nucleus ATAC/RNA sequencing was performed using the DNBelab C Series Single‐Cell ATAC Library Prep Set (MGI, 1000021878) and the DNBelab C Series High‐throughput Single‐Cell Library Preparation Kit (MGI, 940‐000047‐00) following the manufacturers’ instructions. Each chip was loaded with ≈10 000–12 000 nuclei, and 3000‐8000 nuclei were recovered per sample. Sequencing was carried out on the MGI DNBSEQ‐T7 platform, with a minimum depth of 50 000 reads per nucleus for the libraries. Raw sequencing reads were demultiplexed, aligned, and a count matrix or fragment files were generated using DNBelab_C_Series_HT_scRNA‐analysis‐software (https://github.com/MGI‐tech‐bioinformatics/DNBelab_C_Series_HT_scRNA‐analysis‐software). The study utilized the mm10 reference genome for alignment, specifically employing a pre‐messenger RNA reference for nuclear RNA analysis.

### Preprocessing in snRNA Dataset

The filtered_feature_bc_matrix was loaded into R using Seurat (v.4.3.0.1) with the Read10X function. Cells with <200 unique genes and >4000 genes per cell were excluded. Cells were identified as doublets using DoubletFinder (v.2.0.3) and removed excluded from further analysis. The nExp was set to 0.08 × nCells2/10 000, pN was set to 0.25 and pK was set to 0.09.

After the execution of DoubletFinder, the cells remaining from all samples were merged into a single Seurat object. Subsequently, any cells with counts exceeding 10000 per cell or showing more than 20% mitochondrial RNA were excluded. The study then carried out standard normalization and scaling, using a scale factor of 10000 and considering 3000 genes as variable features. This ensured that the expression of all genes was appropriately adjusted. Next, the study applied linear dimensional reduction on the scaled data, focusing on dimensions 1 through 50 and employing a resolution of 0.8. Initially, the major cell types were identified in the snRNA dataset using well‐established marker genes from public datasets. Subsequently, the study honed in on specific subtypes within neurons, oligodendrocytes, microglia, and other major cell types to improve the classification. Re‐clustering was performed for each subset dataset. The study first evaluated the dimensionality of the dataset using function DimHeatmap and ElbowPlot, and then determined the resolution of the dataset using package clustree. Lastly, the study ran a PCA on only cell cycle genes to prevent the effect of cyclic genes on cell clustering. Similarly, cellular subtypes were annotated using classical marker genes. In contrast, for subpopulations of inhibitory neurons that posed challenges in manual annotation, the study relied on published datasets to perform automated annotation using the FindTransferAnchors and TransferData functions. Once the subtypes were defined, the sub‐clustered labels were integrated back into the full atlas.

### Preprocessing in snATAC Dataset

The fragments files were created and loaded into R with the ArchR (v.1.0.2) using the function createArrowFiles. The data were filtered to remove cells with fewer than 1000 unique nuclear fragments per cell or fewer than 4 TSS enrichment score per cell.

The study initially identified doublets using a filterRatio of 1.5 and subsequently removed them using the filterDoublets() function. For the first round of dimensionality reduction and clustering, LSI was employed in ArchR, setting the parameters as follows: iterations at 2, resolution at 0.4, varFeatures at 25 000, dimensions ranging from 1 to 30, and a resolution of 1.7 for clustering. Upon examining the UMAP plots for marker genes, it was observed that clusters 4 and 5 either did not express marker genes or exhibited mixed expression of markers from different cell types. Consequently, the subsetArchRProject function was used to exclude these clusters due to their lower quality.

For the second round of dimensionality reduction and clustering, the study again utilized LSI in ArchR. This time, the study set the iterations at 3, resolutions at 0.1 and 0.2, varFeatures at 50000, dimensions from 1 to 50, and a resolution of 0.8 for addClusters. Moreover, nNeighbors were set at 60 and minDist at 0.5 for addUMAP. Initially, the major cell types were defined in the snATAC dataset using well‐established marker genes from public datasets. Subsequently, the study focused on specific subtypes within neurons, oligodendrocytes, microglia, and other major cell types for further refinement. Re‐clustering was performed for each subset dataset. The label transfer was carried out using function addGeneIntegrationMatrix for labeling the snATAC‐seq with cell types from the snRNA‐seq data. The subtypes were defined and backed sub‐clustered labels to full atlas, and created the pseudo‐bulk replicates which were grouped by subtypes using addGroupCoverages() function. Peak‐calling was then conducted on these pseudo‐bulk replicates on full project using MACS2(v.2.2.8) and these peaks were added to the subproject and generated peak matrix. This concept outlined by Benjamin Ober‐Reynolds et al. aims to enhance the capacity for identifying open chromatin regions that are specific to rare cell subtypes. The addPeak2GeneLinks function in ArchR was used to link gene regulatory elements and gene expression, and high confidence peak‐to‐gene links were established by retaining links with a Pearson correlation coefficient >0.5.

### Cell Type Composition Analysis

To determine whether the subpopulations increase or decrease after injury or therapy, Pertpy (v.0.5.0) was applied to identify significant fluctuations in the compositional data using Bayesian modeling. Initially, the sample and cell type information were extracted from the project using function getCellColData and created a data frame with sample x cell_type format in R. Next, the data were loaded into python and converted into an anndata object using sccoda.util.cell_composition_data module. The resulting object separates the data into the following components: Cell counts are stored in data.X, covariates in data.obs. Then, the MuData object was developed using sccoda_model.load with type at sample_level, generate_sample_level at True, sample_identifier at Sample, covariate_obs at Sample and condition. The cell counts in different subtypes were visualized using the pt.pl.coda.boxplots function, y_scale = “log”. To more intuitively examine the changes in cell type after SCI, the study extracted the data of condition of sham and injury for running MCMC inference, reference_cell_type = ″automatic″ and FDR = 0.05. The study saved the Compositional Analysis summary using write_h5mu function. Finally, the results were imported into R using the read.csv() function for plotting. To analyze the changes in cell types after spinal cord repair, the same method was followed, this time extracting data specific to the therapy condition and injury status.

### GO Analysis

The study conducted Gene Ontology (GO) term enrichment analysis using topGO (version 2.52.0) on the provided dataset. To calculate GO terms for each cluster, gene counts were extracted from the Seurat object using the Get AssayData function and considered genes with at least 2 counts in at least 5 cells as eligible. DEG analysis was carried out using the FindAllMarkers function in Seurat (version 4.3.0.1), and genes with an average log2 fold change greater than 0.5 and an adjusted p‐value less than 0.01 were deemed as interesting genes.

### KEGG Analysis

Gene set enrichment analysis for various genes was performed using clusterProfiler (v.4.9.1). Genes list was created by FindAllMarkers function in Seurat (v.4.3.0.1) and ranking metric by avg_log2FC. The study used gseKEGG function for GSEA, pvalueCutoff = 0.05 and pAdjustMethod = “BH” and visualized the result using cnetplot function.

### Biodistribution of Ce Uptake In Vivo

The biodistribution of Ce uptake in vivo was assessed using inductively coupled plasma mass spectrometry (ICP‐MS). After intraperitoneal injection of either CeO₂ NPs or Ce@UCNP, SCI mice were euthanized at 0.5, 24, and 48 hours post‐injection. Primary organs, including the spinal cord, heart, liver, spleen, lung, and kidney, were collected. These samples were solubilized in aqua regia for ICP‐MS analysis to determine the Ce content.

### Metabolism Assay and BSCB Permeability Assay In Vivo

The metabolism of Ce@UCNP in vivo was detected using a multi‐mode animal live imaging system (AniView100). After intraperitoneal injection of Ce@UCNP, SCI mice were transferred to clean cages, and urine, feces, and blood samples were collected at various time points (0, 1, 2, 4, 6, 8, 12, 16, 24, 36 hours, and 2, 3, 4, 5, 6, 7 days post‐administration). These samples were placed on the imaging platform for up‐conversion imaging, with excitation and emission wavelengths set at 980 nm and 650 nm, respectively, and fluorescence intensity detected at 650 nm.

On the seventh day post‐administration, one hour after injecting FITC‐Dextran into the tail vein of the mice, the mice were euthanized. A 3 cm segment of spinal cord tissue, centered on the injury site, was then surgically collected. This tissue was also placed on the imaging platform for fluorescence imaging, with excitation and emission wavelengths set at 480 nm and 520 nm, respectively, and fluorescence intensity detected at 520 nm.

### Apoptosis Assay

Apoptosis was detected using flow cytometry. bEnd.3 cells were seeded into 6‐well culture plates and incubated with H_2_O_2_‐enriched medium for 12 hours. After removing the medium, the cells were cultured in media containing varying concentrations of CeO_2_ NPs and Ce@UCNP for another 12 hours. The cells were then washed with PBS, trypsinized, and collected into a 15 ml centrifuge tube. The cells were centrifuged at 5000 rpm for 5 minutes. The supernatant was discarded, and the cells were resuspended in 500 µL of PBS. The suspension was transferred to a 1.5 ml centrifuge tube and centrifuged again at 5000 rpm for 5 minutes. The supernatant was removed, and 300 µL of Binding Buffer, 2.5 µL of Annexin V‐FITC, and 2.5 µL of PI were added to the cell pellet. The mixture was incubated in the dark for 15 minutes. After another centrifugation at 5000 rpm for 5 minutes, the supernatant was aspirated, and 300 µL of PBS was added. The sample was transferred to a flow‐sampling tube for fluorescence analysis using the FACS Array Bioanalyzer (BD Biosciences). Apoptosis was analyzed and plotted using FlowJo 7.6 software.

### Permeability Assay & Morphology and Number Assay

The permeability assays utilize the Fluorescein isothiocyanate dextran fluorescent probe (FITC‐Dextran, MW 4000, MedChemExpress) and the bEnd.3 cell line, which consists of endothelial cells isolated from mouse brain tissue with endothelioma. The bEnd.3 cells were seeded in transwell inserts of 12‐well culture plates at a density of 1 × 10⁴ cells/cm^2^. Each group was prepared in triplicate. After 48 hours of culture, the cells grew adherently, formed tight junctions in the upper chamber, and maintained the medium level in the upper chamber above that of the lower chamber for 12 hours. Subsequently, the cells were incubated in a medium enriched with H₂O₂ for 12 hours to disrupt the BSCB. The medium was then discarded, and the cells were cultured in a medium containing specific concentrations of CeO₂ NPs and Ce@UCNP for 24 hours. Cells were washed with PBS, and medium containing FITC‐Dextran (0.1 mg mL^−1^) was added to the upper chamber. Samples from the lower chamber were collected after 15 minutes. The fluorescence signal was measured by zymography (Ex = 485 nm, Em = 538 nm) and permeability was calculated. Data were analyzed and plotted using GraphPad Prism 7.

Moreover, the morphology and number of bEnd.3 cells in the upper chamber were assessed using immunofluorescence staining. Cells were digested with trypsin from each upper chamber and collected into 15 mL centrifuge tubes. The tubes were centrifuged for 5 minutes at 5000 rpm, and the supernatant was removed. Each centrifuge tube was then filled with 200 µL medium to resuspend the cells, which were subsequently planted on cell crawls in 24‐well plates. After 6 hours, precooled 4% paraformaldehyde was added to each well for 45 minutes at 4 °C. The paraformaldehyde was removed, and the cells were washed with PBS. The cell membrane was permeabilized through three consecutive 10‐minute treatments with TBST containing 1% Triton X‐100. The cells were blocked for an hour at room temperature using 3% bovine serum albumin in TBST with 1% Triton X‐100, then incubated with an anti‐βIII tubulin antibody (Abcam, ab18207) at 4 °C for 12 hours. The cells were subsequently washed three times with TBST containing 1% Triton X‐100, each wash lasting 10 minutes. They were then cultured with anti‐mouse Alexa Fluor 488 (Abcam, ab150073) for 2 hours. The cells were washed three times and treated with a mounting solution containing DAPI (EMS, Hatfield, PA). Finally, microscopic examination and image analysis were conducted using microscopes (Carl Zeiss, Germany).

## Conflict of Interest

The authors declare no conflict of interest.

## Author Contributions

K.W., J.Z., R.L., and T.C. contributed equally to this work. Z.J., H.L., C.L., and Y.L. designed the project and experiments. J.Z., W.L., and M.Z. involved in synthesis and characterization of Ce@UCNP‐BCH. K.W., T.C., Y.M., P.W., J.L., and J.Z. performed cells and animal experiments and the data analysis. R.L. and Y.Y. conducted snATAC‐seq experiments, while X.L. and Y.W. carried out snRNA‐seq experiments. W.M. maintained and improved the DNBelab_C_Series_HT_scRNA‐analysis‐software. R.L. analyzed the snATAC dataset and the snRNA dataset. K.W., R.L., J.Z., and Z.J. wrote the manuscript. Z.J., H.L., J.Z., and Y.L. provided the funds for this research. Z.J., H.L., C.L., and Y.L. participated in the supervision of this research. Z.J., H.L., C.L., Y.L., R.L., J.Z., and K.W. revised the manuscript. All authors have read and approved the final manuscript. The remaining authors declare no competing interests.

## Supporting information



Supporting Information

## Data Availability

The data that support the findings of this study are available from the corresponding author upon reasonable request.
