# Correction to “Single‐Cell Multi‐Omics Assessment of Spinal Cord Injury Blocking via Cerium‐Doped Upconversion Antioxidant Nanoenzymes”

**DOI:** 10.1002/advs.202509430

**Published:** 2026-02-09

K. Wang, J. Zheng, R. Li, T. Chen, Y. Ma, P. Wu, J. Luo, J. Zhu, W. Lin, M. Zhao, Y. Yuan, W. Ma, X. Lin, Y. Wang, L. Liu, P. Gao, H. Lin, C. Liu, Y. Liao, Z. Ji, Single‐Cell Multi‐omics Assessment of Spinal Cord Injury Blocking via Cerium‐doped Upconversion Antioxidant Nanoenzymes. *Adv. Sci*. **2025**, 12, 2412526.

https://doi.org/10.1002/advs.202412526

In Figure S15 (Supporting Information), the “Sham” image was mistakenly chosen from the group of “Injury + Ce@UCNP‐BCH” during the layout of the images. As the conclusions of the H&E staining result of primary organs were based on the correct images, the conclusions remain unchanged.

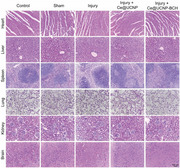

We apologize for this error.

