# Supplementary material for: Single‐Cell Multi‐omics Assessment of Spinal Cord Injury Blocking via Cerium‐doped Upconversion Antioxidant Nanoenzymes
Source: Adv Sci (Weinh). 2025 Jan 9;12(8):2412526. doi: 10.1002/advs.202412526 (PMC11848599; doi:10.1002/advs.202412526)
Supplement: Supplementary file 1 — Supporting Information [file ADVS-12-2412526-s001.docx]

**Supplementary Material**

**Single-Cell Multi-omics Assessment of Spinal Cord Injury Blocking via Cerium-doped Upconversion Antioxidant Nanoenzymes**

**Supplementary Method**

**Data Conversion**

To convert ArchR project to Seurat object, we extracted GeneScoreMatrix from the ArchR object using the *getMatrixFromProject* function in ArchR and the gene names were assigned as row names. The GeneScoreMatrix is built by associating chromatin accessibility data with gene annotations, calculating activity scores based on accessibility near gene regulatory regions. We then converted it into a Seurat assay object using *CreateAssayObject* function and wrapped in a Seurat object using *CreateSeuratObject* function. To convert Seurat object to anndata, we firstly saved Seurat object in the Seurat format (.h5Seurat) using the *SaveH5Seurat* function and then converted it to the annData format (.h5ad) using the *Convert* function from the SeuratDisk^[1]^ package (v0.0.0.90).

**Preprocessing in snATAC/snRNA dataset**

In this study, the snATAC dataset was analysed mainly using the ArchR^[2]^ package (v1.0.2), and the snRNA dataset was analysed mainly by the Seurat^[3]^ package (v4.3.0.1). Complex heatmap was created and customized using ComplexHeatmap^[4]^ package (v2.18.0). We identified differentially expressed genes (DEGs) between two groups of cells using *FindMarkers* function with default parameters (Wilcoxon Rank Sum test) and volcano plots are generated for each pairwise comparison using EnhancedVolcano^[5]^ package (v1.20.0). To leverage the more flexible visualization capabilities of the Scanpy^[6]^ package (v1.9.3), we converted the ArchR project into the anndata format and performed dot plot and heatmap visualizations in Python (v3.10.14).

**Sankey diagram** **Generation**

We used the networkD3^[7]^ (v0.4) packages to create a Sankey diagram for visualizing the distribution of cell subtypes across experimental conditions and cell types in the snATAC dataset. Metadata is processed to calculate percentages for each transition, and nodes are assigned custom colors for better differentiation. The diagram illustrates the flow of cells across categories, providing an intuitive visualization of cell population dynamics and compositional differences.

**Confusion matrix heatmap** **Generation**

We generated a confusion matrix heatmap to compare snATAC-seq predicted groups and snRNA-seq clusters using the Jaccard Index to quantify similarity. The matrix is reordered based on predefined cluster orders, with annotated rows and columns for ATAC and RNA labels and a white-to-purple gradient representing similarity scores. The heatmap, created using the ComplexHeatmap package, visually assesses the concordance between clusters, aiding in evaluating the integration of ATAC and RNA datasets.

**Comparison of neuron proportions**

We generated boxplots and performed Wilcoxon tests to compare the proportions of neurons in different locations (“Mid,” “Ventral,” “Dorsal”) across experimental conditions (“S,” “I,” “C”) in the snATAC-seq dataset. Sample with over 50 cells are included for analysis, significant differences are assessed with corrected p-values (Bonferroni), and results are visualized with jittered data points and annotated significance levels (e.g., ns for not significant, * for p ≤ 0.05, ** for p ≤ 0.01, *** for p ≤ 0.001, and **** for p ≤ 0.0001).

**Condition-specific differential abundance of cell neighborhoods**

We used the miloR^[8]^ package (v1.10.0) to analyze differential abundance (DA) of cell neighborhoods in snATAC-seq dataset, leveraging graph-based representations for spatial relationships and focusing on condition-specific enrichment. We firstly converted an ArchR project into a SingleCellExperiment object, with count data (PeakMatrix), dimensionality reductions (LSI, UMAP), and metadata. A K-Nearest Neighbor (KNN) graph was built with *buildGraph* function using the LSI dimensions (reduced.dim = "LSI") and parameters such as k = 20 (number of neighbors) and d = 30 (number of dimensions). Neighborhoods are defined with *makeNhoods* function, specifying a cell proportion (prop = 0.05) and refining neighborhoods to improve representativeness. A design matrix for DA testing was created using condition and Sample metadata. DA analysis was performed with *testNhoods* function, applying the design formula (~ 0 + condition), the contrast ("conditionS - conditionI"), and FDR weighting (fdr.weighting = "graph-overlap") to account for neighborhood overlap. Neighborhood graphs were constructed with *buildNhoodGraph* function and visualized on the UMAP layout using *plotNhoodGraphDA* function, where DA significance is mapped to node size and color (alpha = 0.1, size_range = c(1, 4), node_stroke = 0.1). Neighborhoods were annotated with cell types using *annotateNhoods* fucntion, and mixed neighborhoods are excluded by setting a threshold (Clusters_fraction < 0.6). Final DA results are summarized using beeswarm plots with *plotDAbeeswarm* function, grouping neighborhoods by their dominant cell type.

**Peak-to-Gene Linking**

We began by adding motif annotations to the project with *addMotifAnnotations* function, using the “cisbp” motif database to enable downstream motif analysis. To investigate correlations between peak accessibility and gene expression, the *plotPeak2GeneHeatmap* function is employed, grouping cells by cell subtypes and clustering peaks with k-means (k=5, same as cell subtypes). Next, we got the peak-to-gene association matrices (returnMatrices=TRUE) and processes them to extract peak and gene information. Using the *getPeakSet* and *getMatches* functions, all peaks and their corresponding motif matches were retrieved and formatted for analysis. Peaks were grouped into clusters based on k-means clustering results, and motif enrichment was computed for each cluster using ArchR computeEnrichment , with results formatted into a SummarizedExperiment object. The motif enrichment data was normalized and visualized as a heatmap, where rows represent TFs annotated with their maximum enrichment scores and columns represent cell subtypes. To further explore DNA sequence motifs, we generated sequence logos using the *seqLogo* function of seqLogo^[9]^ package (v1.68.0).

To visualizing peak-to-gene associations and RNA expression levels across conditions in snATAC-seq dataset, integrating chromatin accessibility and gene regulation, we began by getting all peaks using *getPeakSet* function and annotating motifs with *getPositions* function. Selected motifs, such as *Klf7*, *Sp1*, and *Egr1*, were identified as marker regions. Promoters linked to genes of interest were extracted, and only peak-to-gene loops with correlations above a threshold (corCutOff=0.3) are retained using *getPeak2GeneLinks* function. The genomic regions of interest were bracketed around these loops and resized for visualization. Next, *plotBrowserTrack* function was used to create tracks for chromatin accessibility, including bulk peak tracks, feature annotations, loop connections, and gene annotations. This visualization highlights the regulatory relationships between peaks and genes across conditions (C, I, S). To explore RNA expression, selected genes (*Apod*, *Scd2*, and *Olig1*) were analyzed using integrated RNA expression data from the Gene Integration Matrix retrieved with *getMatrixFromProject* function. Expression values were stratified by condition, and Wilcoxon rank-sum tests were performed to assess differential expression with Bonferroni-adjusted p-values. The results were visualized with violin and boxplots using ggplot2^[10]^ package (v3.5.0), where p-values and statistical significance are annotated.

**References**

[1] H. Paul, S. Rahul, *https://github.com/mojaveazure/seurat-disk* **2023**.

[2] J. M. Granja, M. R. Corces, S. E. Pierce, S. T. Bagdatli, H. Choudhry, H. Y. Chang, W. J. Greenleaf, *Nature Genetics* **2021**, 53, 403.

[3] T. Stuart, A. Butler, P. Hoffman, C. Hafemeister, E. Papalexi, W. M. Mauck, 3rd, Y. Hao, M. Stoeckius, P. Smibert, R. Satija, *Cell* **2019**, 177, 1888.

[4] Z. Gu, *iMeta* **2022**, 1, e43.

[5] K. Blighe., S. Rana., E. Turkes., B. Ostendorf., A. Grioni., M. Lewis., *https://doi.org/10.18129/B9.bioc.EnhancedVolcano* **2023**.

[6] F. A. Wolf, P. Angerer, F. J. Theis, *Genome biology* **2018**, 19, 15.

[7] J. J. Allaire., C. Gandrud., K. Russell., C. Yetman., *https://CRAN.R-project.org/package=networkD3* **2017**.

[8] E. Dann, N. C. Henderson, S. A. Teichmann, M. D. Morgan, J. C. Marioni, *Nature Biotechnology* **2022**, 40, 245.

[9] O. Bembom., R. Ivanek., *https://doi.org/10.18129/B9.bioc.seqLogo* **2023**.

[10] H. Wickham, *ggplot2*, Springer International Publishing, **2016**.


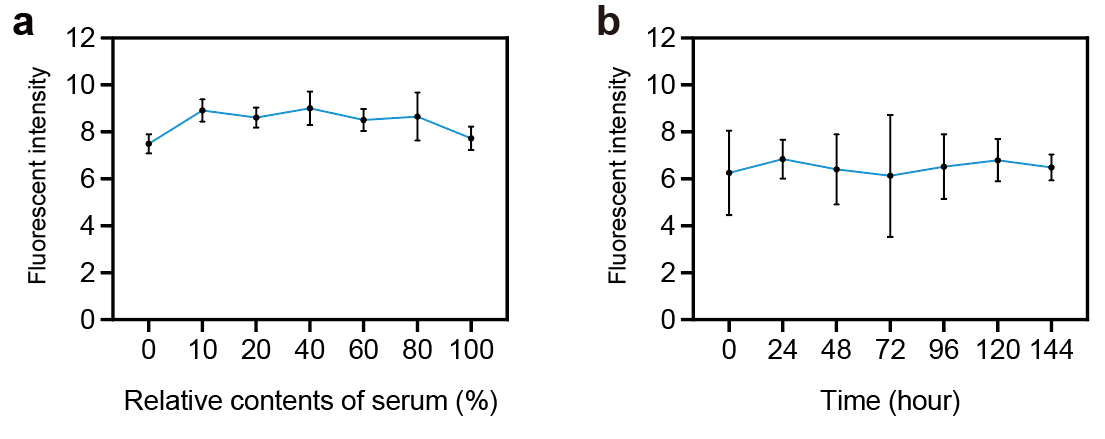


**Figure S1. Changes in upconversion luminescence of Ce@UCNP-BCH in PBS (pH =7.4) with (a) different serum concentration (from 0 to 100%)and (b) 100% serum keeping for various time (from 0 to 144 h)** (n=3)**.**


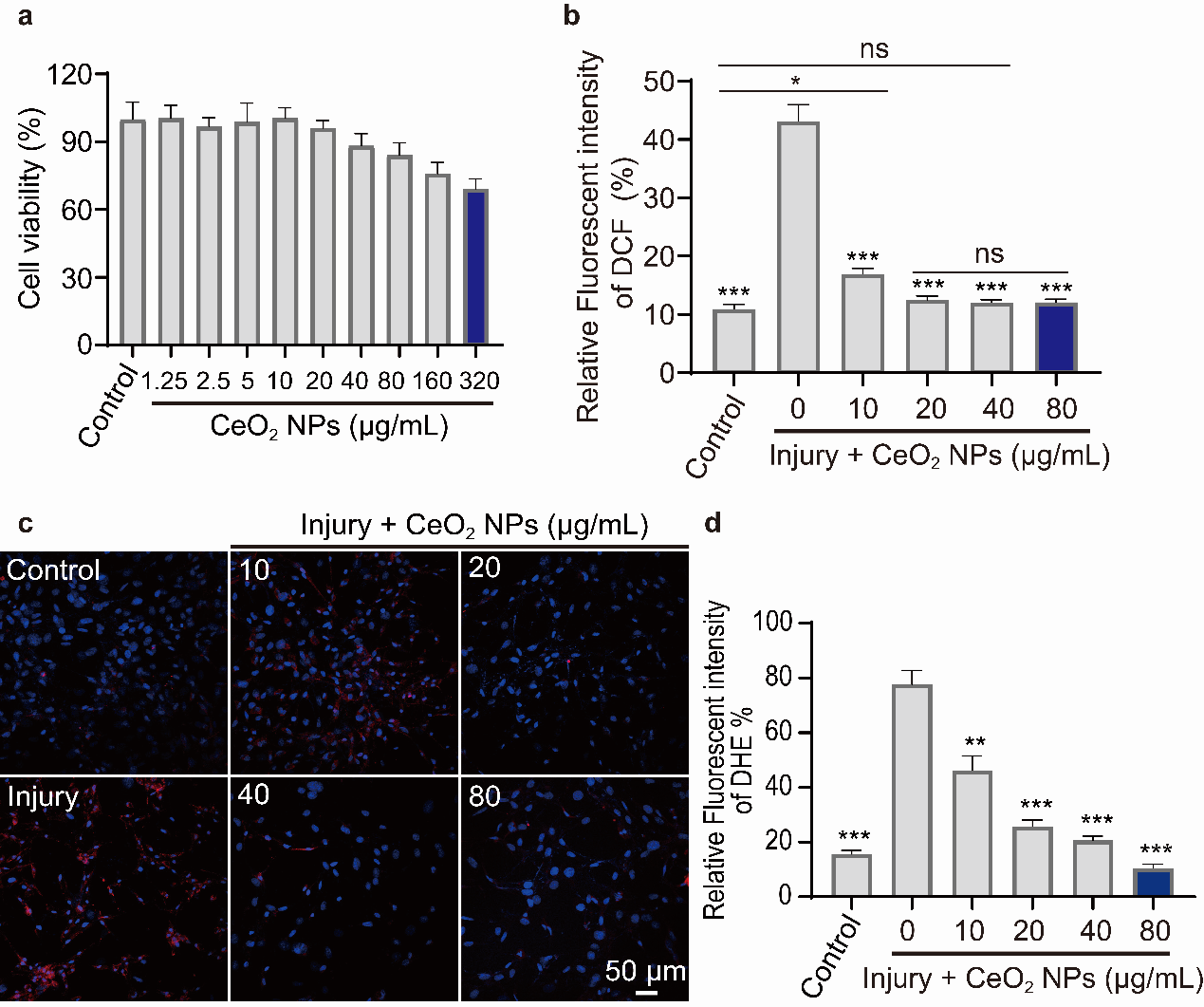


**Figure S2.** **ROS-scavenging performance of CeO_2_ NPs *in vitro*.** **a**. Cell viability was tested by Cell Counting Kit-8 (CCK-8) in HT-22 incubated with different concentration of CeO_2_ NPs (n=6). **b**. Quantity analysis of intensity of the DCFH-DA staining in HT-22 cells. Intracellular ROS production in HT-22 cell tested by DHE (n=6). **c**. Representative images of DHE staining of HT-22 cells treated with the different concentration of CeO_2_ NPs. **d**. Quantity analysis of intensity of the DHE staining in HT-22 cells for **c** (n=6). * means p < 0.05, ** means p < 0.01, *** means p < 0.001, ns means not significant.

**
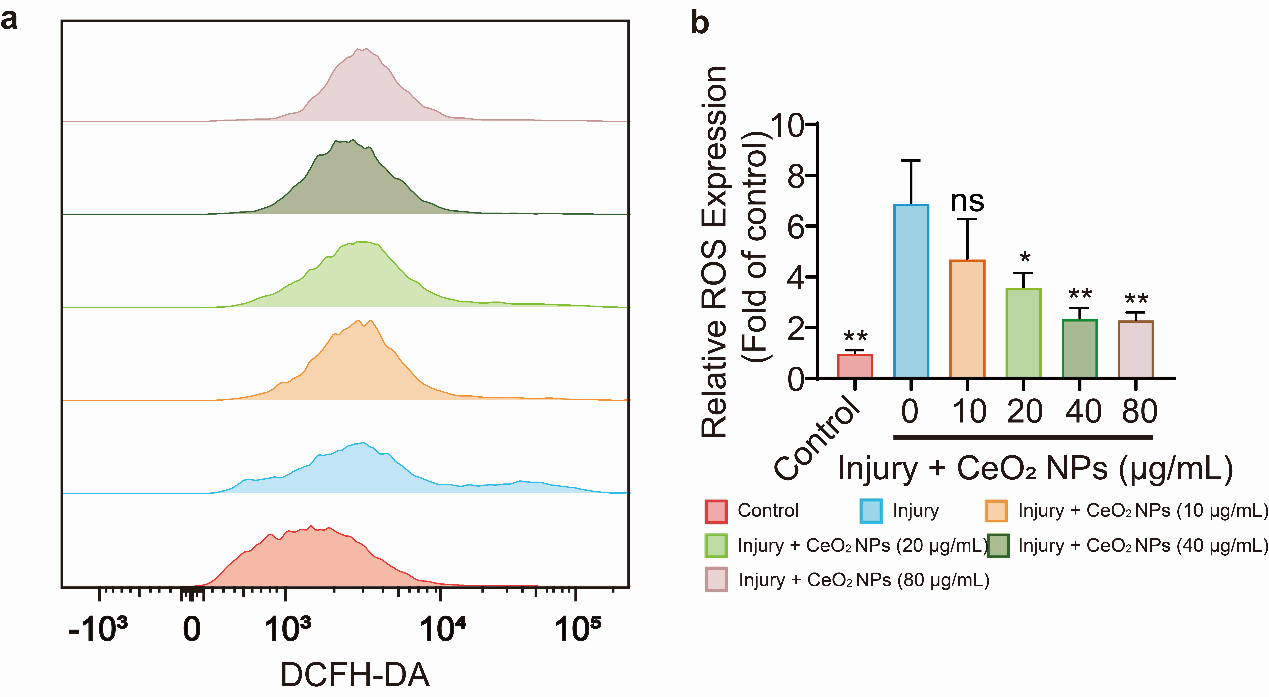
**

**Figure S3.** **Intracellular ROS level of HT-22 cells which were detected by flow cytometry using DCFH-DA Assay Kit.** Analysis **a** and quantification **b** of the relative ROS expression (n=3). * means p < 0.05, ** means p < 0.01, *** means p < 0.001, vs injury group, and ns means not significant.


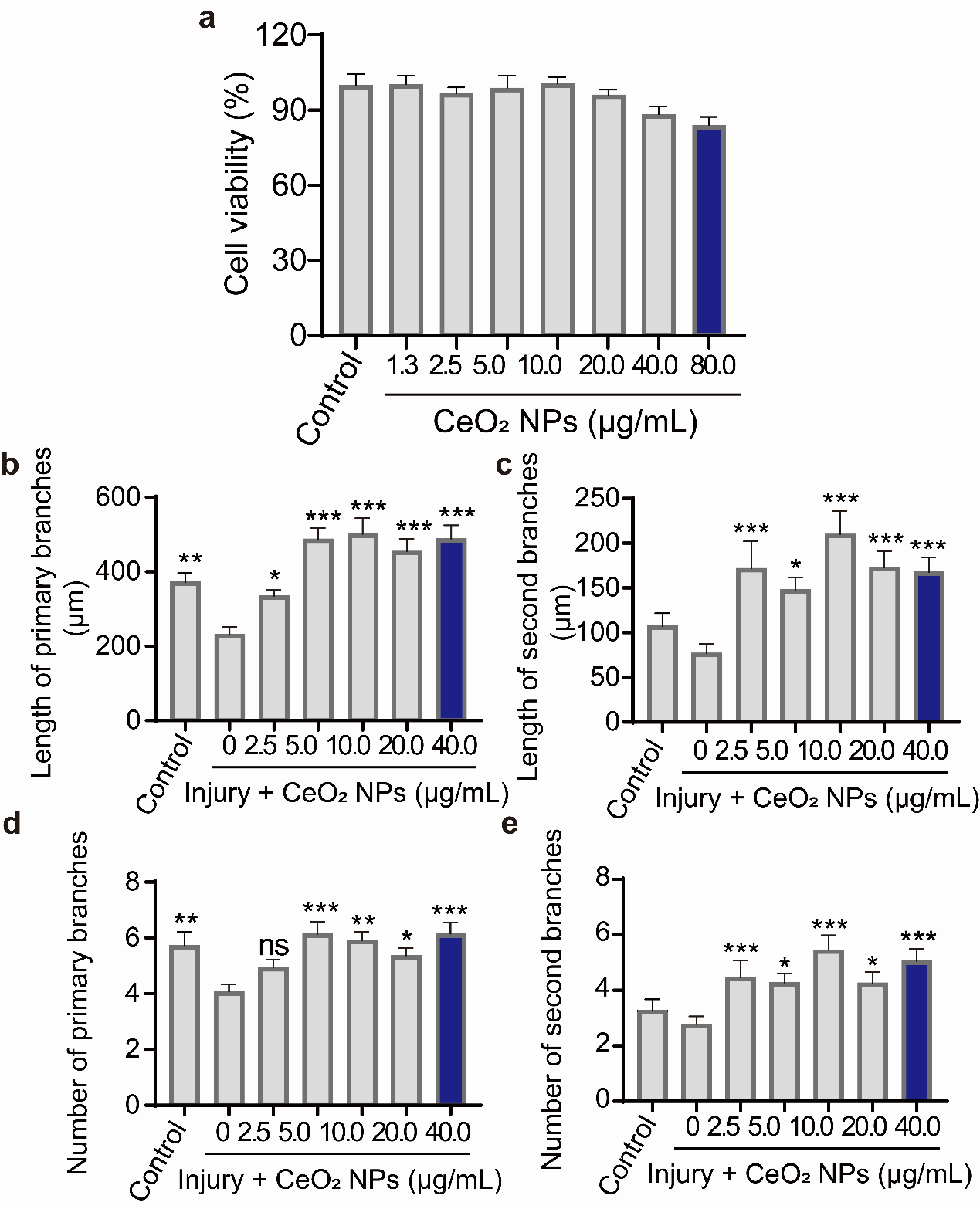
**Figure S4. Restoration of neural behaviors through the utilization of CeO_2_ NPs. a**. Cell viability was tested by CCK-8 in hippocampal neurons incubated with different concentration of CeO_2_ NPs (n=6). Quantified relative length of neuronal primary **b** and second **c** branches, and number of neuronal primary **d** and second **e** branches in damaged hippocampal neurons treated with the different concentration of CeO_2_ NPs (n=30). * means p < 0.05, ** means p < 0.01, *** means p < 0.001, vs injury group, and ns means not significant.


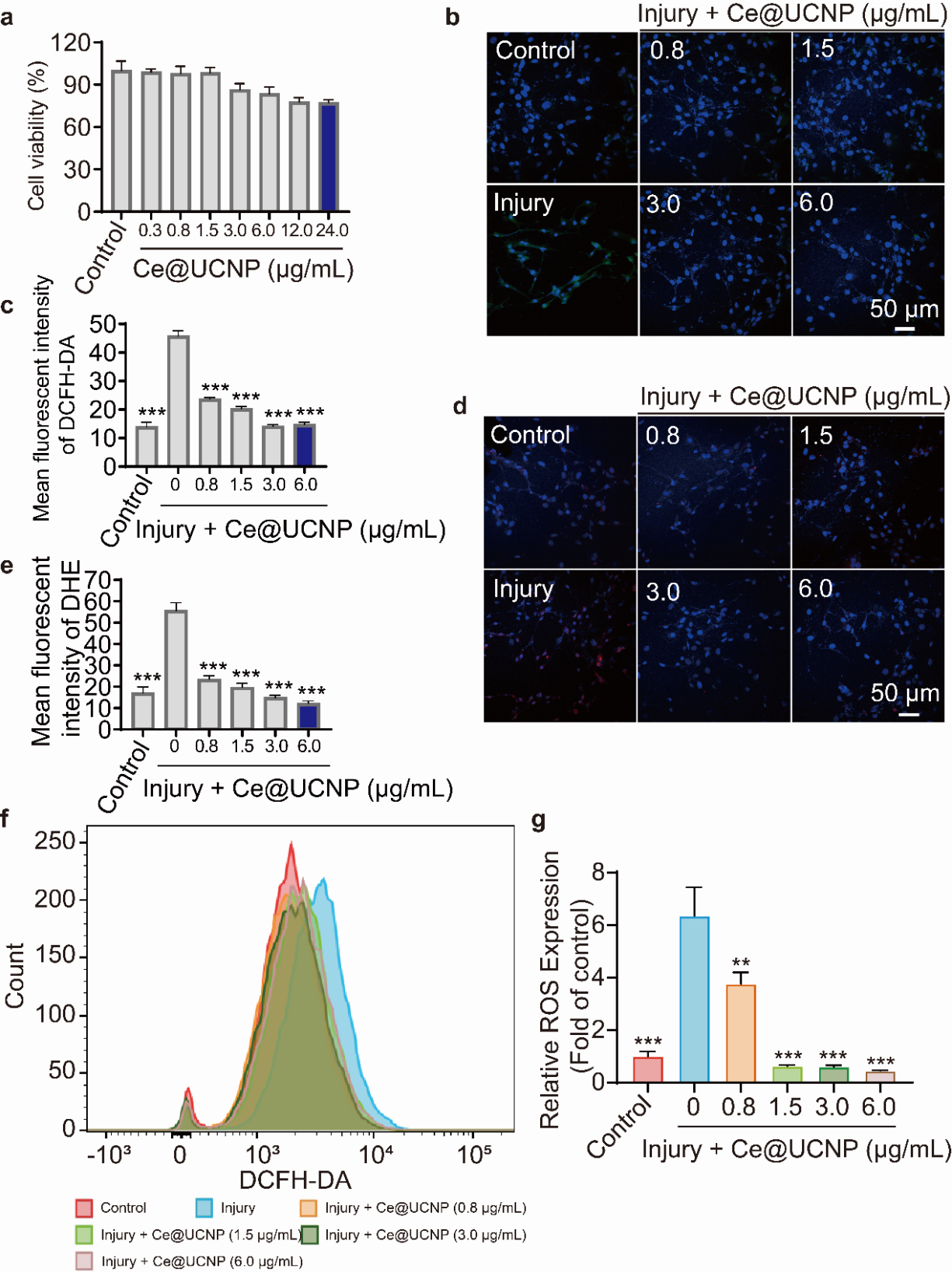


**Figure S5. ROS-scavenging performance of Ce@UCNP *in vitro*. a**. Cell viability was tested by CCK-8 in HT-22 cells incubated with different concentration of Ce@UCNP (n=3). **b**. Representative images of DCFH-DA staining of HT-22 cells treated with the different concentration of Ce@UCNP. **c**. Quantity analysis of intensity of the DCFH-DA staining in HT-22 cells for **b** (n=6). **d**. Representative images of DHE staining of HT-22 cells treated with the different concentration of Ce@UCNP. **e**. Quantity analysis of intensity of the DHE staining in HT-22 cells for **d** (n=6). Intracellular ROS level of HT-22 cells which were detected by flow cytometry using DCFH-DA Assay Kit. Analysis **f** and quantification **g** of the relative ROS expression (n=3). * means p < 0.05, ** means p < 0.01, *** means p < 0.001, vs injury group, and ns means not significant.


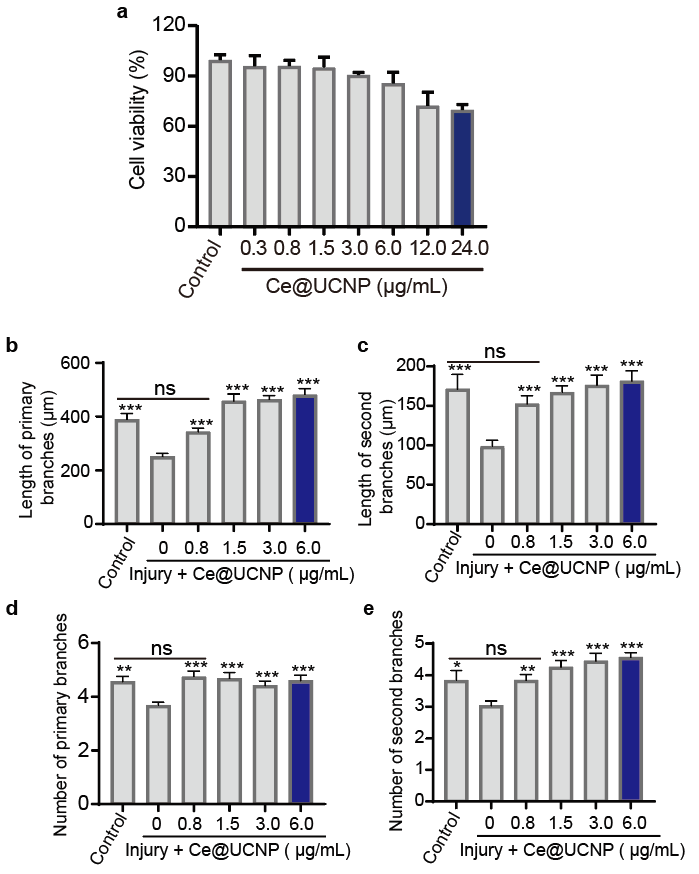


**Figure S6. Restoration of neural behaviors through the utilization of Ce@UCNP. a**. Cell viability was tested by CCK-8 in hippocampal neurons incubated with different concentration of Ce@UCNP (n=3). Quantified relative length of neuronal primary **b** and second **c** branches, and number of neuronal primary **d** and second **e** branches in damaged hippocampal neurons treated with the different concentration of Ce@UCNP (n=30). * means p < 0.05, ** means p < 0.01, *** means p < 0.001, vs injury group, and ns means not significant.


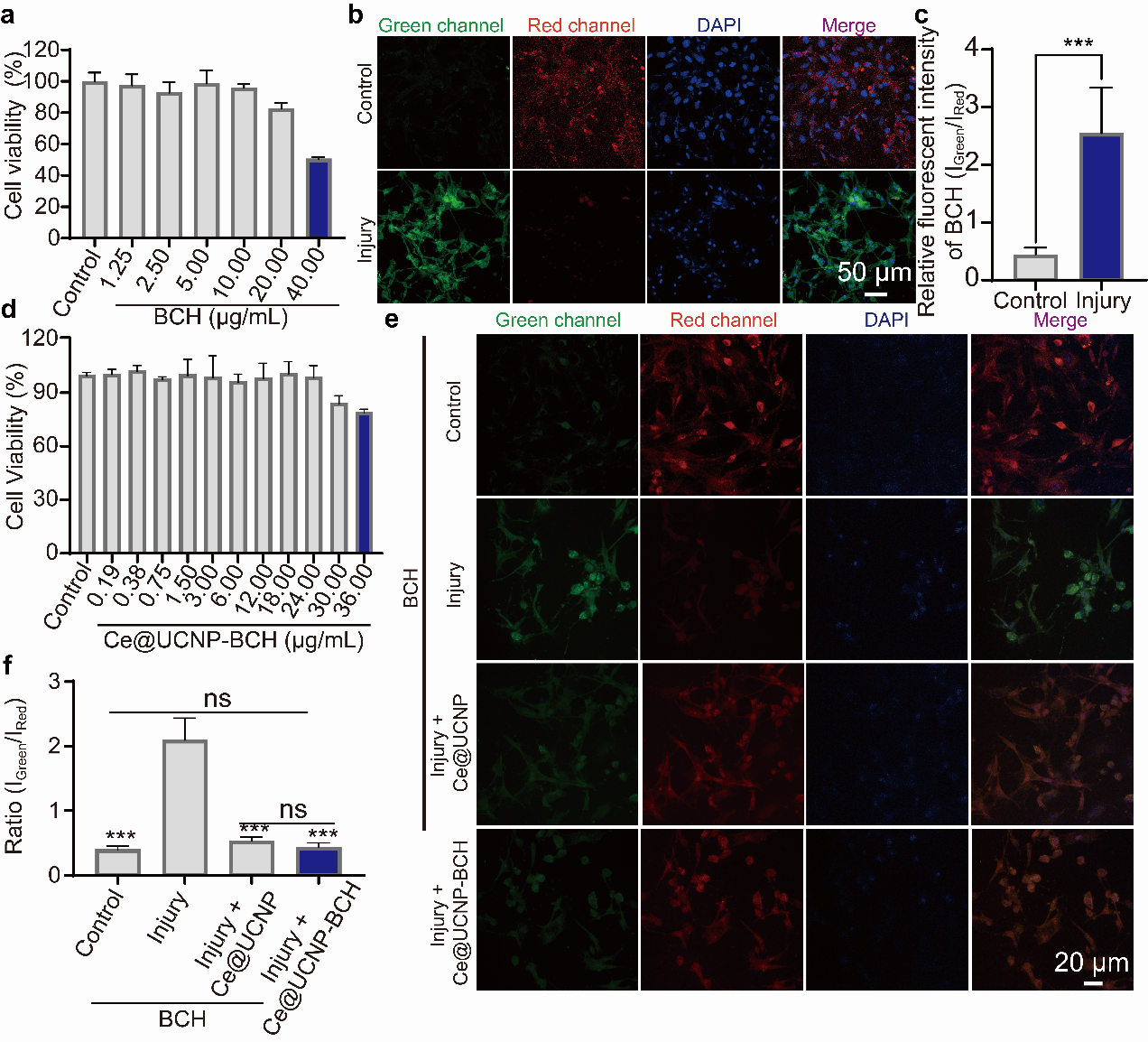


**Figure S7.** **Fluorescence behaviors of Ce@UCNP-BCH *in vitro*. a**. Cell viability was tested by CCK-8 in HT-22 incubated with different concentration of BCH (n=6). **b**. Fluorescence behaviors of BCH. **c**. Quantified relative fluorescent intensity of BCH for **b** (n=6). **d**. Cell viability was tested by CCK-8 in HT-22 incubated with different concentration of Ce@UCNP-BCH (n=6). **e**. Fluorescence behaviors of Ce@UCNP-BCH. **f.** Quantified relative fluorescent intensity of Ce@UCNP-BCH for **e** (n=6). * means p < 0.05, ** means p < 0.01, *** means p < 0.001, ns means not significant.


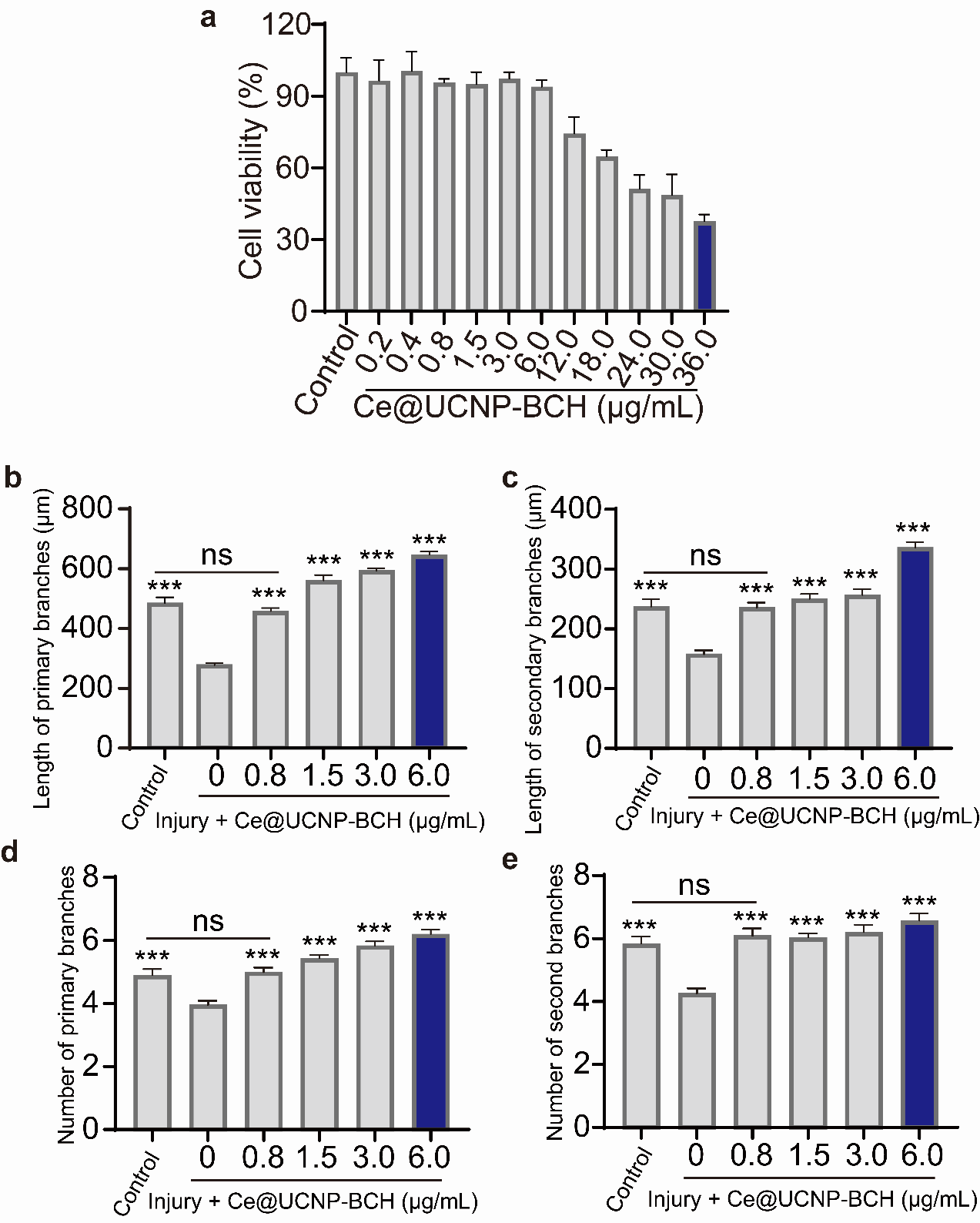


**Figure S8.** **Restoration of neural behaviors through the utilization of Ce@UCNP-BCH. a**. Cell viability was tested by CCK-8 in hippocampal neurons incubated with different concentration of Ce@UCNP-BCH (n=6). Quantified relative length of neuronal primary **b** and second **c** branches, and number of neuronal primary **d** and second **e** branches in damaged hippocampal neurons treated with the different concentration of Ce@UCNP-BCH (n=30). * means p < 0.05, ** means p < 0.01, *** means p < 0.001, vs injury group, and ns means not significant.


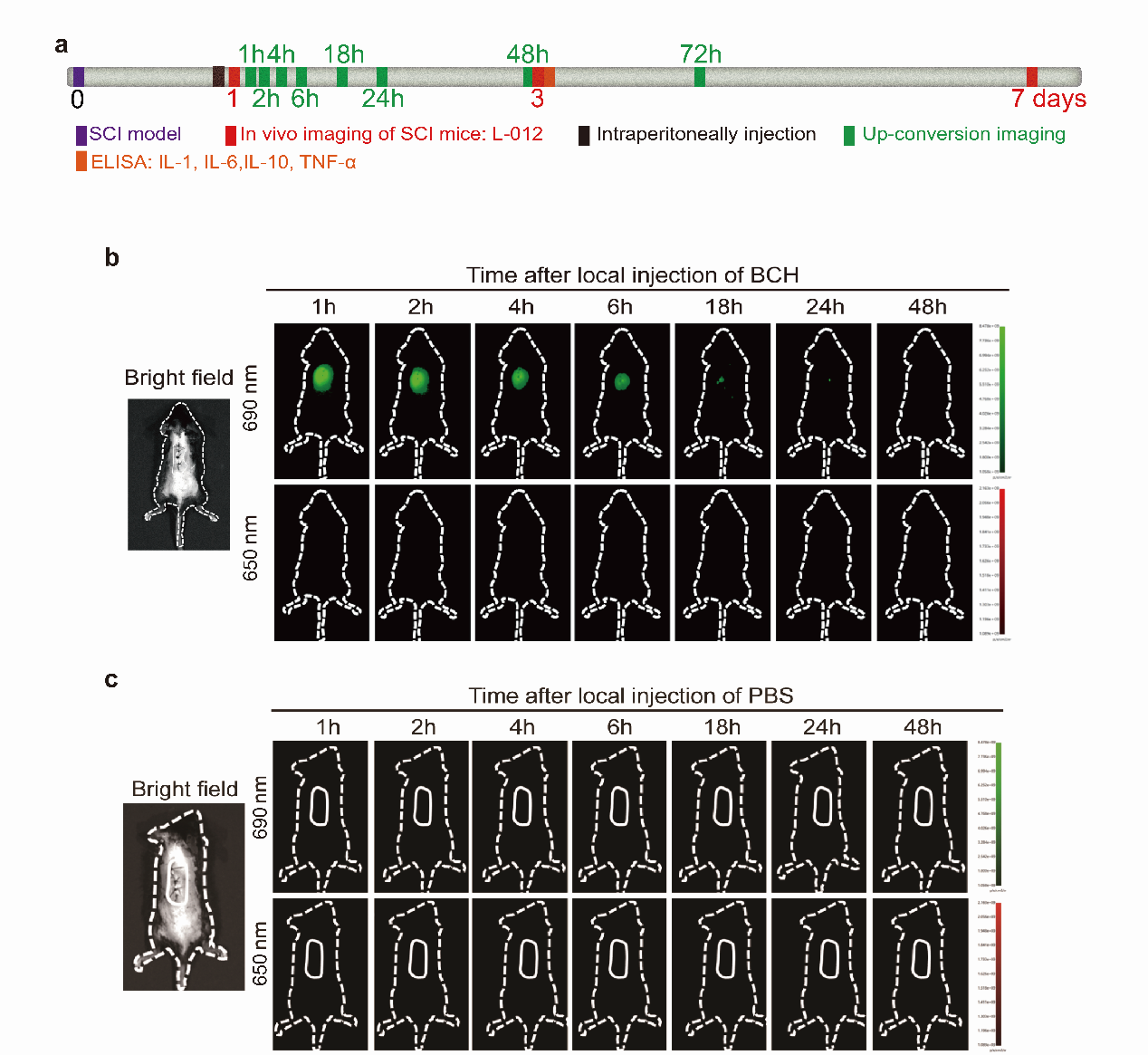
**Figure S9. Experimental schedule and fluorescence behaviors of BCH *in vivo*. a**. Experimental schedule of SCI, materials administration, mouse imaging in vivo. **b**. In vivo living UCL imaging of mouse intraperitoneally injected BCH. **c.** In vivo living UCL imaging of mouse intraperitoneally injected PBS.


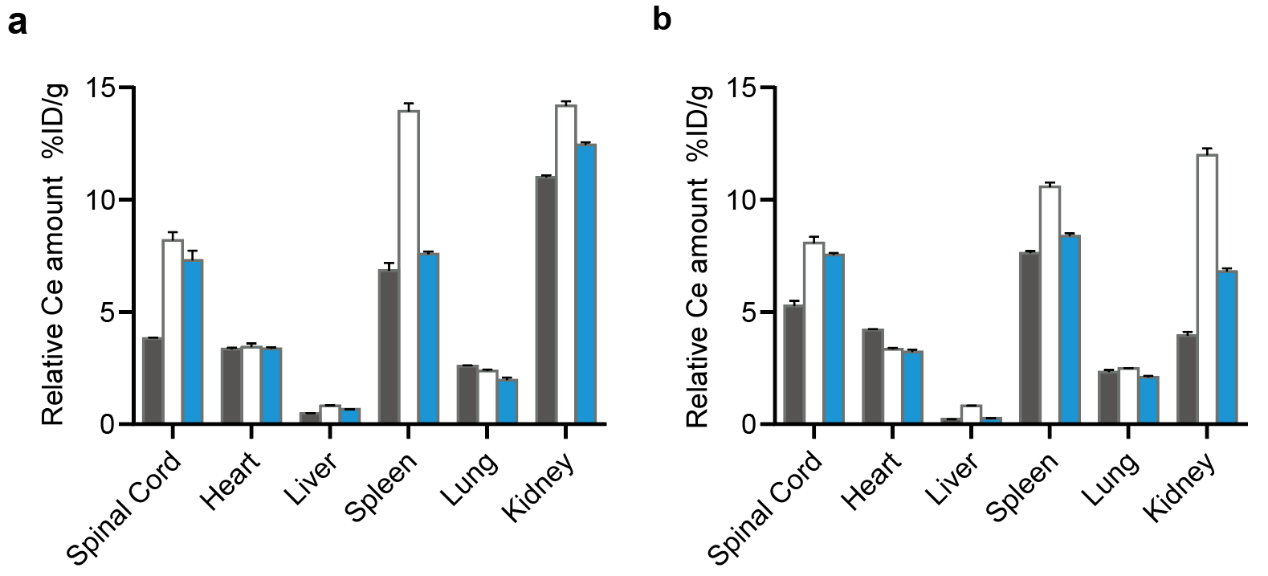


**Figure S10. Biodistribution of Ce uptake in the spinal cord, heart, liver, spleen, lung, and kidney at 0.5, 24 and 48 hours post-injection.** Primary organs ICP-MS of mouse injected CeO_2_ NPs (**a**) or Ce@UCNP(**b**) (n=5).


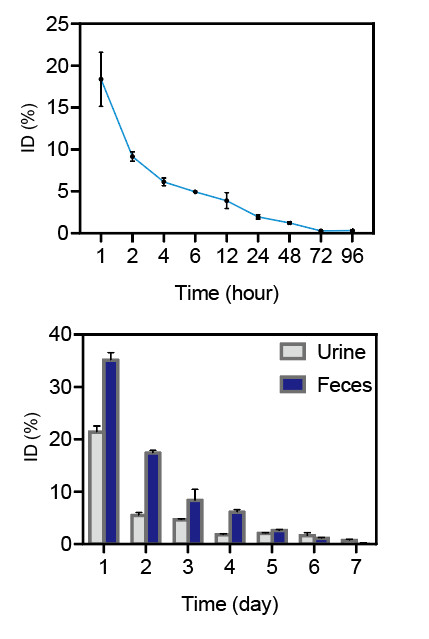


**Figure S11. The blood circulation of Ce@UCNP** (n=5)**.**

**
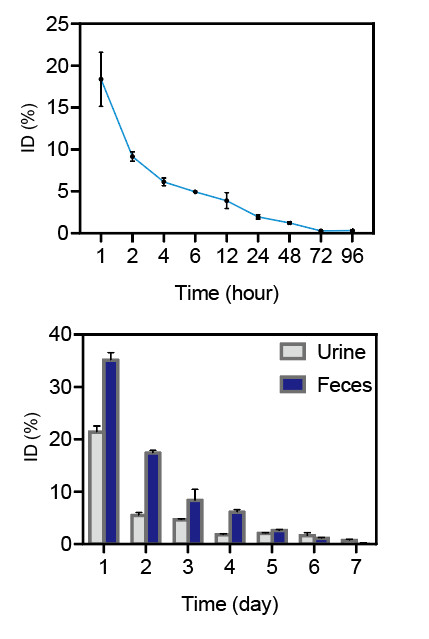
**

**Figure S12. The excretion profile (urine and feces) of Ce@UCNP** (n=5)**.**


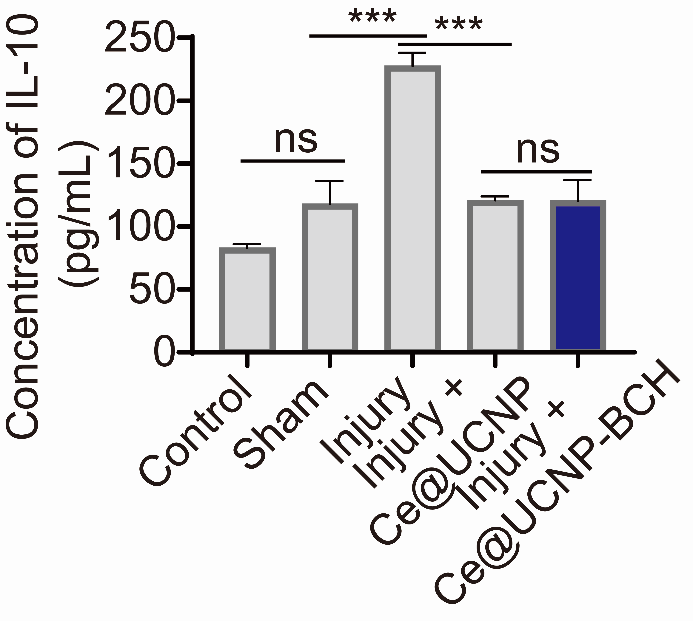


**Figure S13. The expression levels of IL-10 in the SCI mouse after treatments (n=3).** * means p < 0.05, ** means p < 0.01, *** means p < 0.001, ns means not significant.


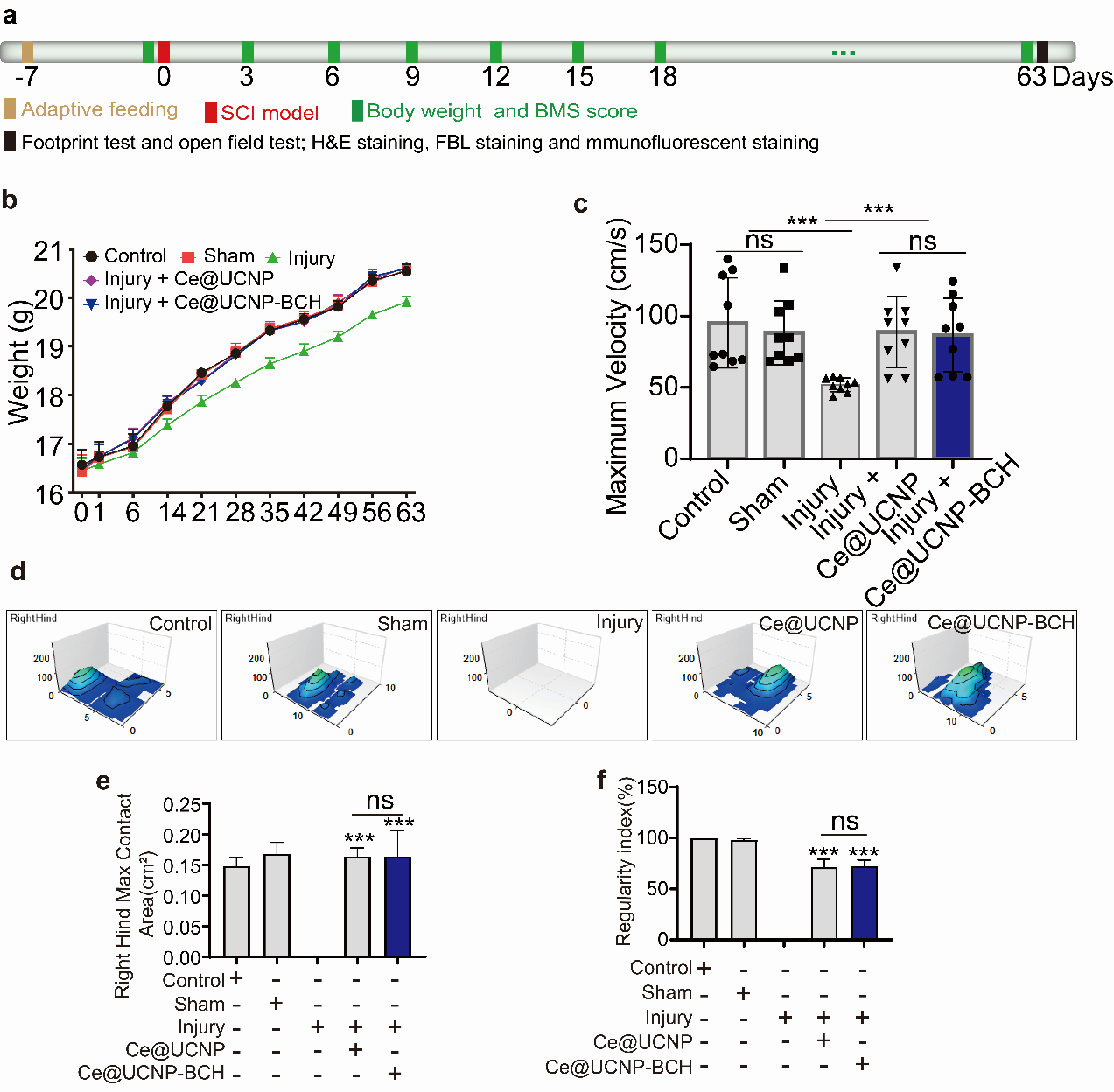


**Figure S14. Experimental schedule and therapeutic effect of Ce@UCNP-BCH *in vivo*. a**. Experimental schedule of Ce@UCNP-BCH treatment. **b**. The body weight of mouse in all groups were assessed within 63 days of post SCI (n=9). **c**. Open field test was also applied to evaluate the motor function recovery of the mouse with the parameters being mean maximum velocity (n=9). Catwalk test was also applied to evaluate the motor function recovery of the mouse with the parameters being mean 3D footprint intensities **d**, Hindlimb max contact area **e** and regularity index **f** (n=3). * means p < 0.05, ** means p < 0.01, *** means p < 0.001, ns means not significant.


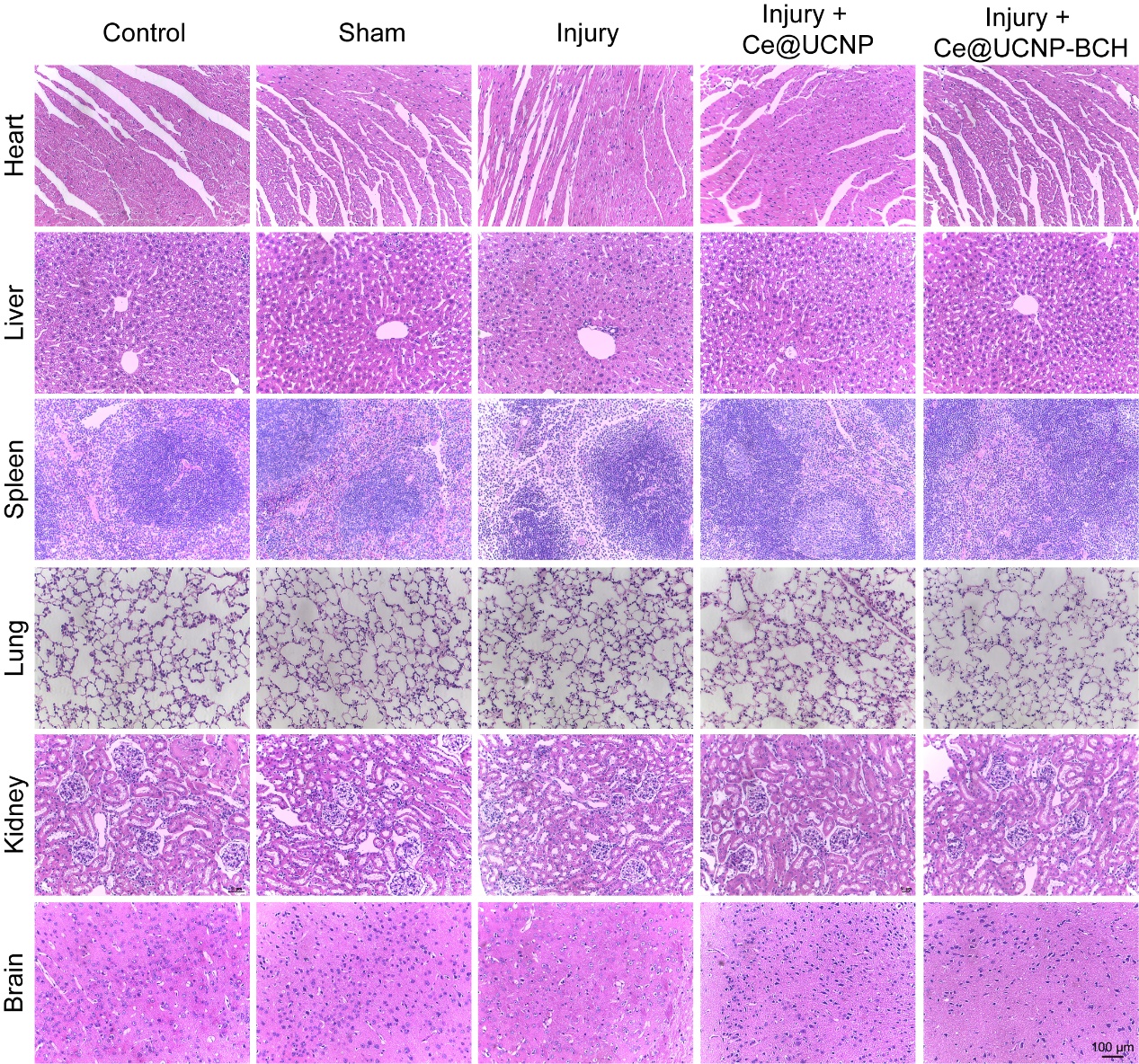


**Figure S15. The H&E staining images of excised heart, liver, spleen, lung, kidney and Brain from different groups including control, sham, injury, injury+Ce@UCNP, and injury + Ce@UCNP-BCH group, scale bar =100 μm.**


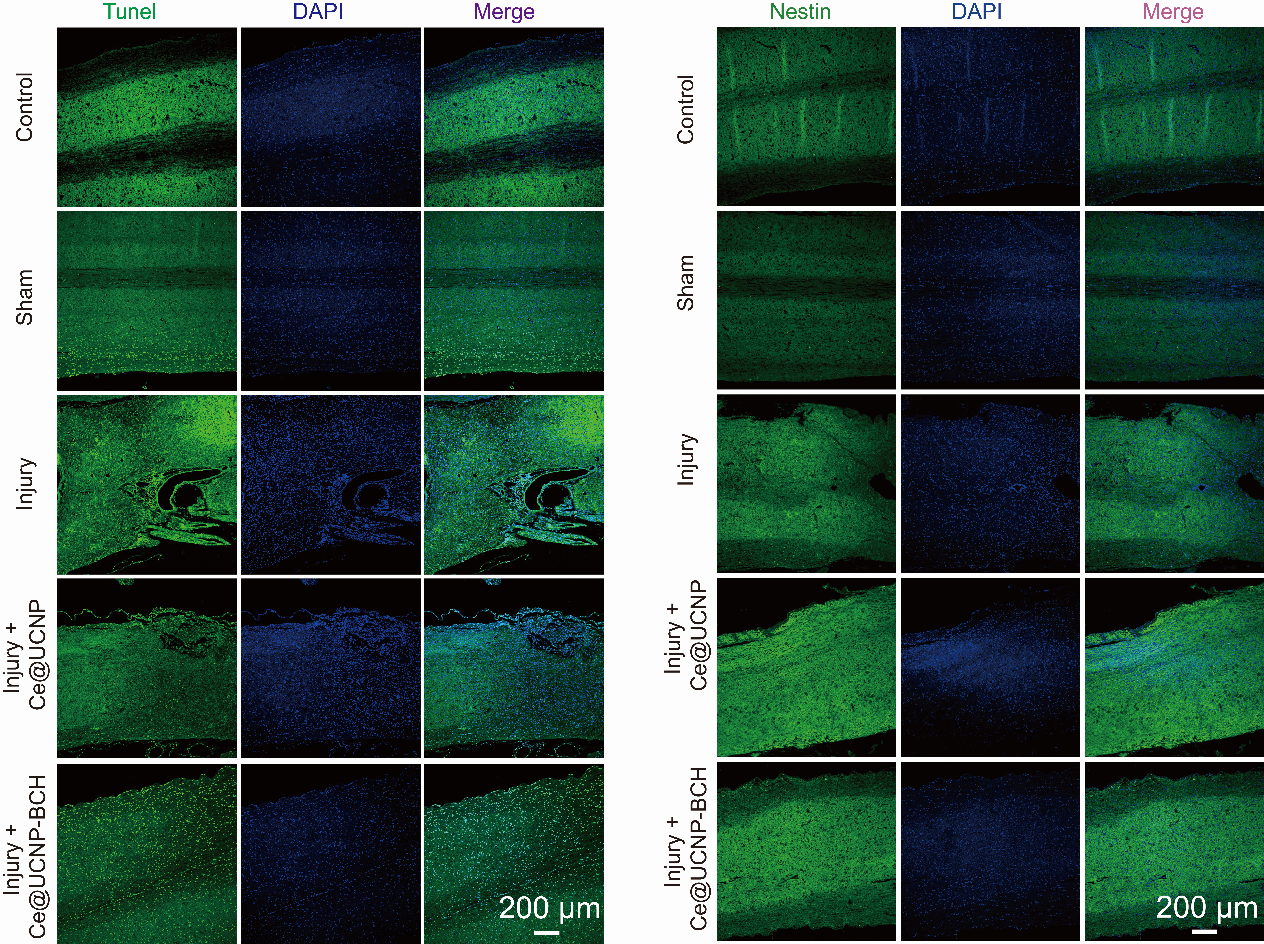


**Figure S16. Immunofluorescence images of spinal cords stained using anti-Tunel and anti-Nestin. a**. Immunofluorescence images of spinal cords stained using anti-Tunel, scale bar = 200 μm. **b**. Immunofluorescence images of spinal cords stained using anti-Nestin, scale bar = 200 μm.


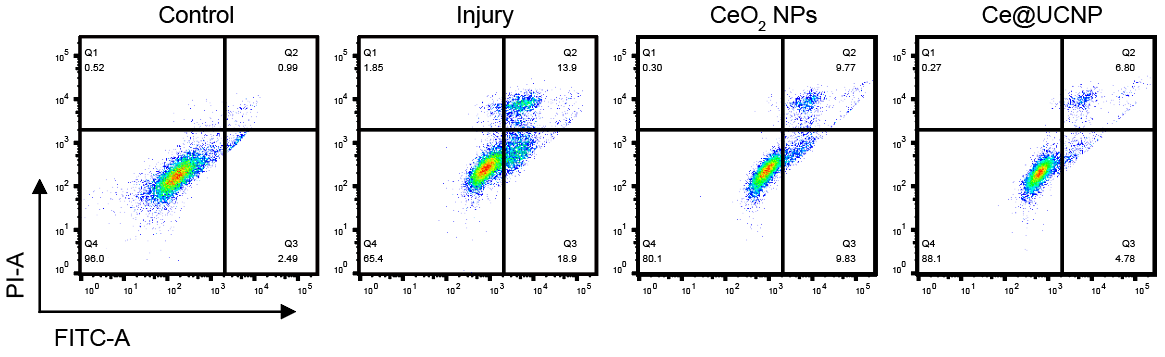


**Figure S17. In vitro detecting cell death with different treatments as assessed by flow cytometry.**


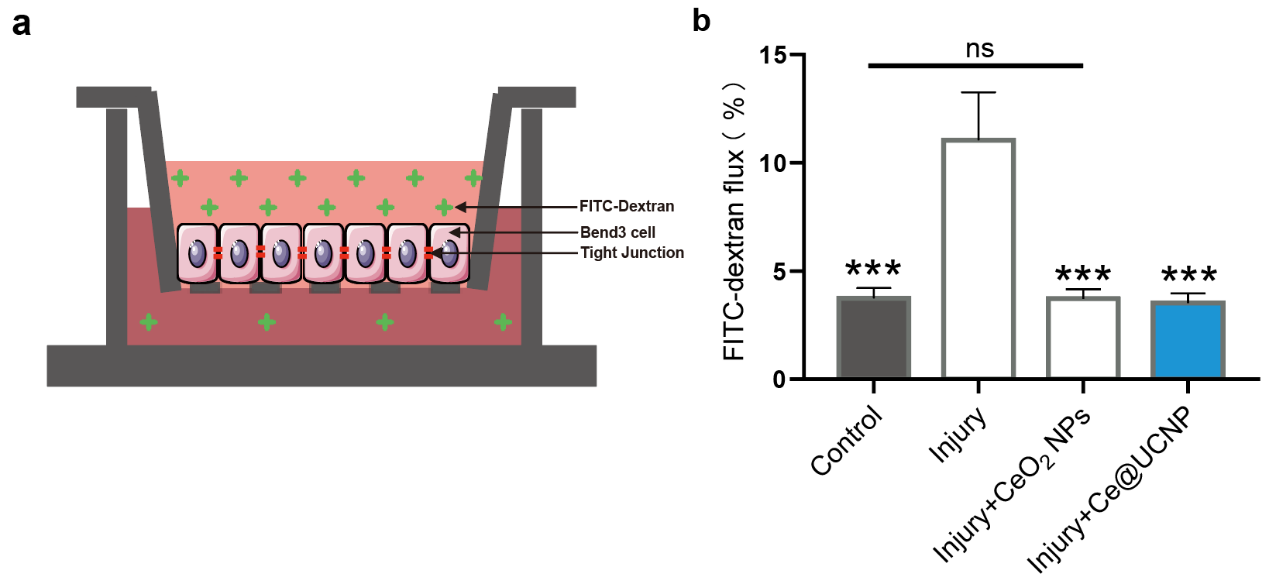


**Figure S18. Transwell permeation assay with different treatments. a**. Overview of the Transwell permeation assay. **b**. Quantity analysis of FITC-dextran flux with different treatments in bEnd.3 cells (n=4). * means p < 0.05, ** means p < 0.01, *** means p < 0.001, vs injury group, and ns means not significant.


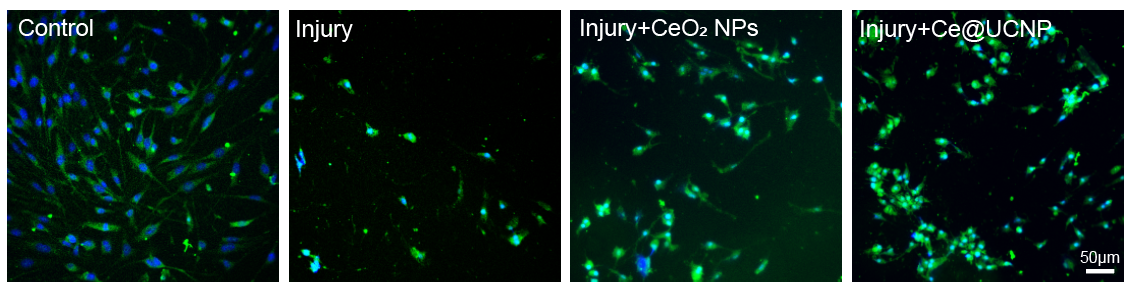


**Figure S19. Microscopy fluorescence images of bEnd.3 under different treatments,** Scale bar = 50 µm.


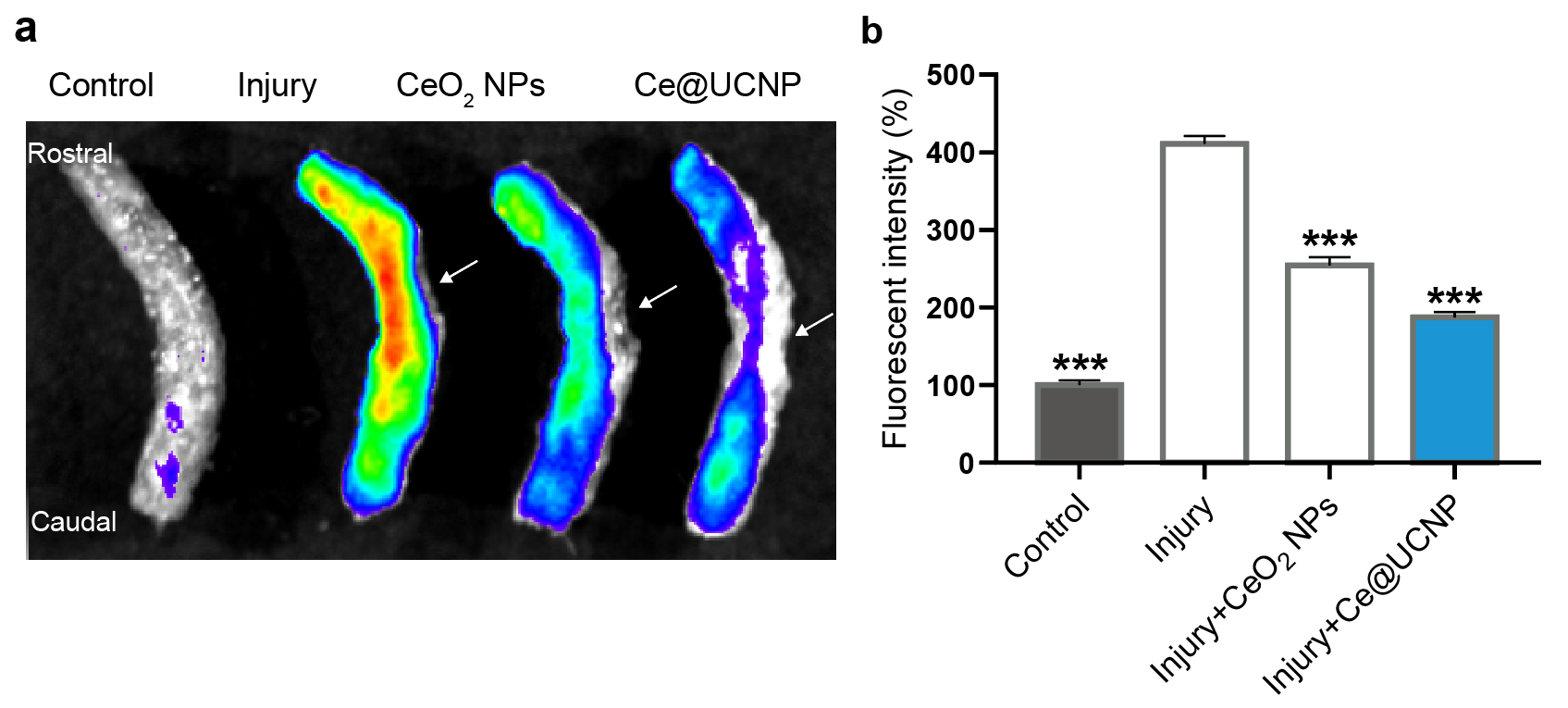


**Figure S20. BSCB permeation assay *in vivo*. a.** Representative images of FITC-Dextran-stained spinal cord injury site after different treatments. **b.** Quantitative analysis of the intensity of fluorescence in spinal cord injury site (n = 3). * means p < 0.05, ** means p < 0.01, *** means p < 0.001, vs injury group, and ns means not significant.


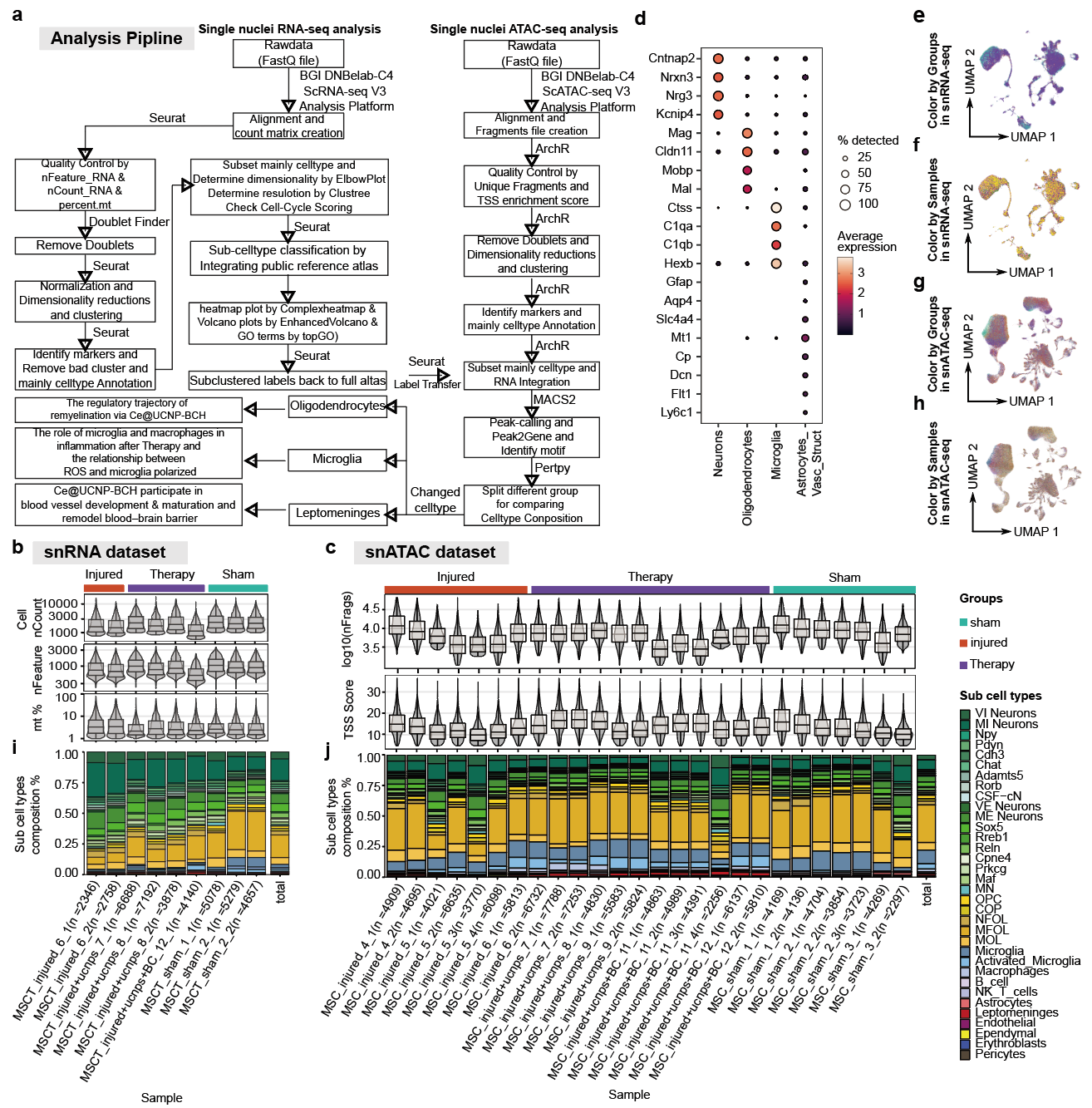


**Figure S21. Quality control of single nuclei datasets a.** Schematic of the computational workflow for snATAC-seq and snRNA-seq. **b.** Violin plots of the number of UMIs sequenced and the number of RNA genes and the percent of mitochondrial RNA from each sample in snRNA-seq. **c.** Violin plots of the log10(unique nuclear fragments) and TSS-enrichments for each sample in snATAC-seq. **d.** Dot plot showed the marker genes for each major cell type of snRNA-seq, the size of the dot corresponds to the percentage of cells expressing the genes in each cell type, the color represents the average expression level. **e.** UMAP plot of spinal cord nuclei of snATAC-seq, colored by condition of snRNA-seq. **f.** UMAP plot of spinal cord nuclei of snRNA-seq, colored by samples of snRNA-seq. **g.** UMAP plot of spinal cord nuclei of snATAC-seq, colored by condition of snATAC-seq. **h.** UMAP plot of spinal cord nuclei of snATAC-seq, colored by samples of snATAC-seq. **i.** Stacked bar plot showing the fraction of all sample composed of each cell subtype for snRNA-seq, the total proportions for each cell subtype are shown in the rightmost column**. j.** Same as g, but for the all sample in snATAC-seq.


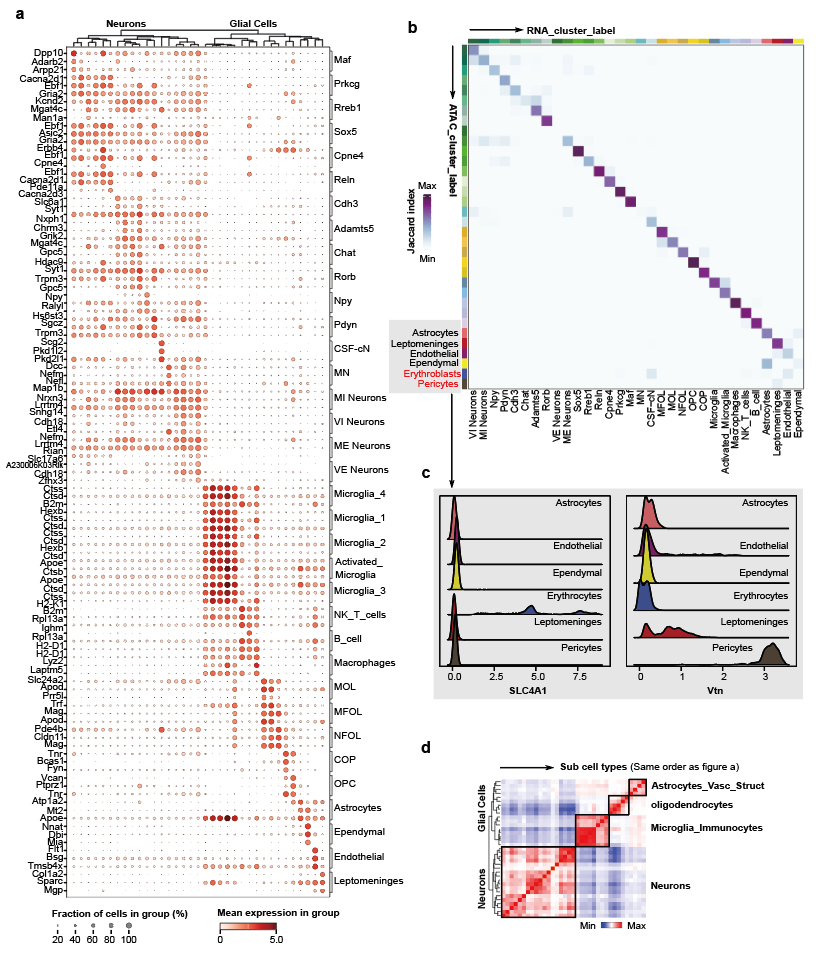


**Figure S22. Annotation of single nuclei datasets a.** Dot plot showed the top three genes for each cell type of snRNA-seq, the size of the dot corresponds to the percentage of cells expressing the genes in each cell type, the color represents the average expression level. **b.** Correspondence between snATAC-seq and snRNA-seq cluster label in the full dataset. **c.** Ridges plot showed gene score of selected marker genes of Erythroblasts and Pericytes within Astrocytes_Vasc_Struct in snATAC-seq. **d.** Pearson correlations between Sub cell types based on RNA expression.


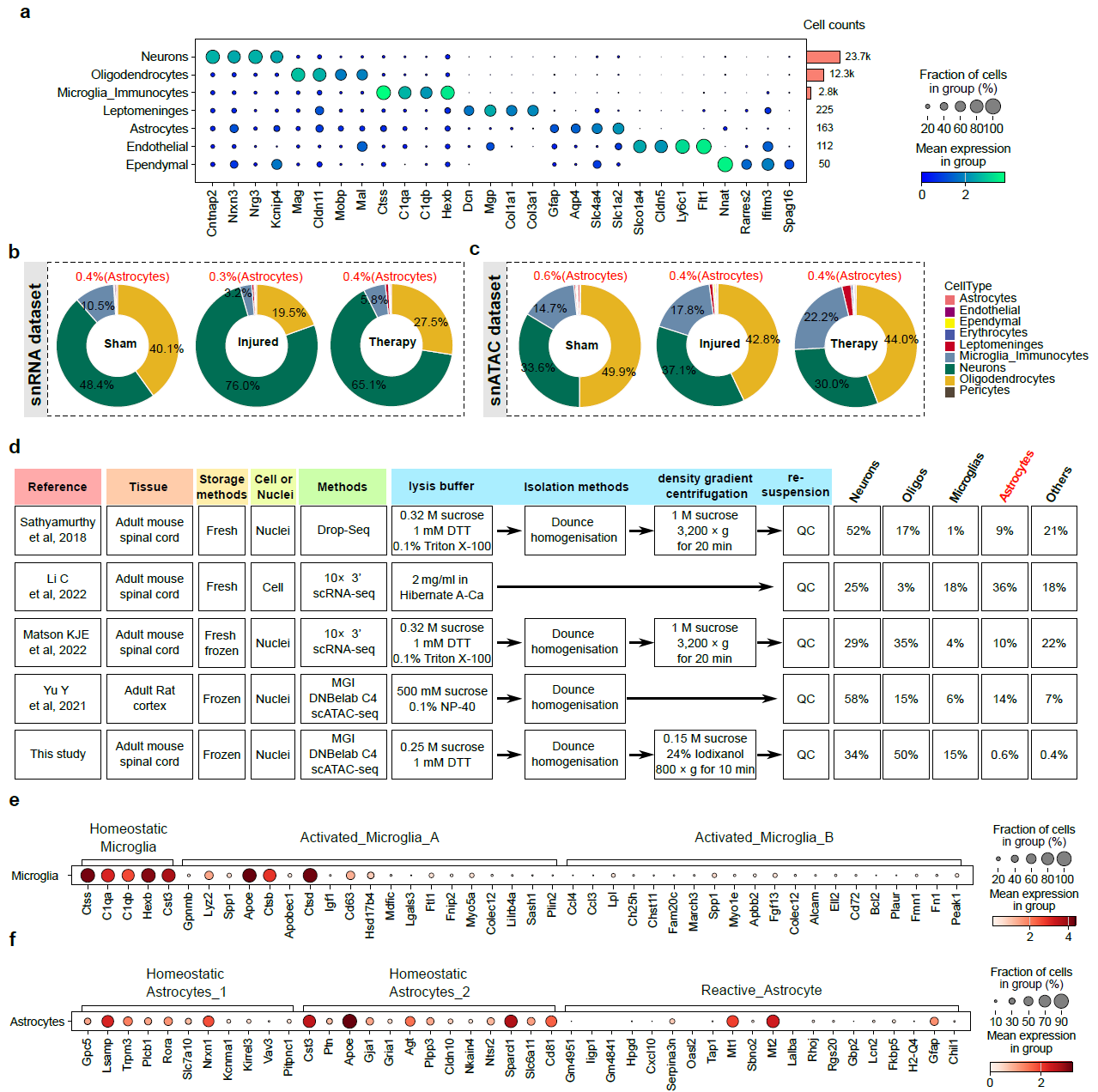


**Figure S23. Assessing Cell Type Composition in Spinal Cord Tissue. a.** Dot plot showed the marker genes for each major cell type of snRNA-seq, the number of cells of each cell type is shown as a bar graph on the right. **b.** Pie chart illustrates the proportion of various cell types in the sham, injured, and therapy groups from the snRNA-seq dataset. **c.** Same as b, but for snATAC-seq dataset. **d.** Table compares different studies based on their methodology. Table also includes the observed cell type proportions from each study, highlighting differences in cell type capture efficiency. **e.** Dot plot highlights the expression of marker genes across different microglia subtypes in the snRNA-seq dataset. **f.** Same as e, but for different astrocytes subtypes.


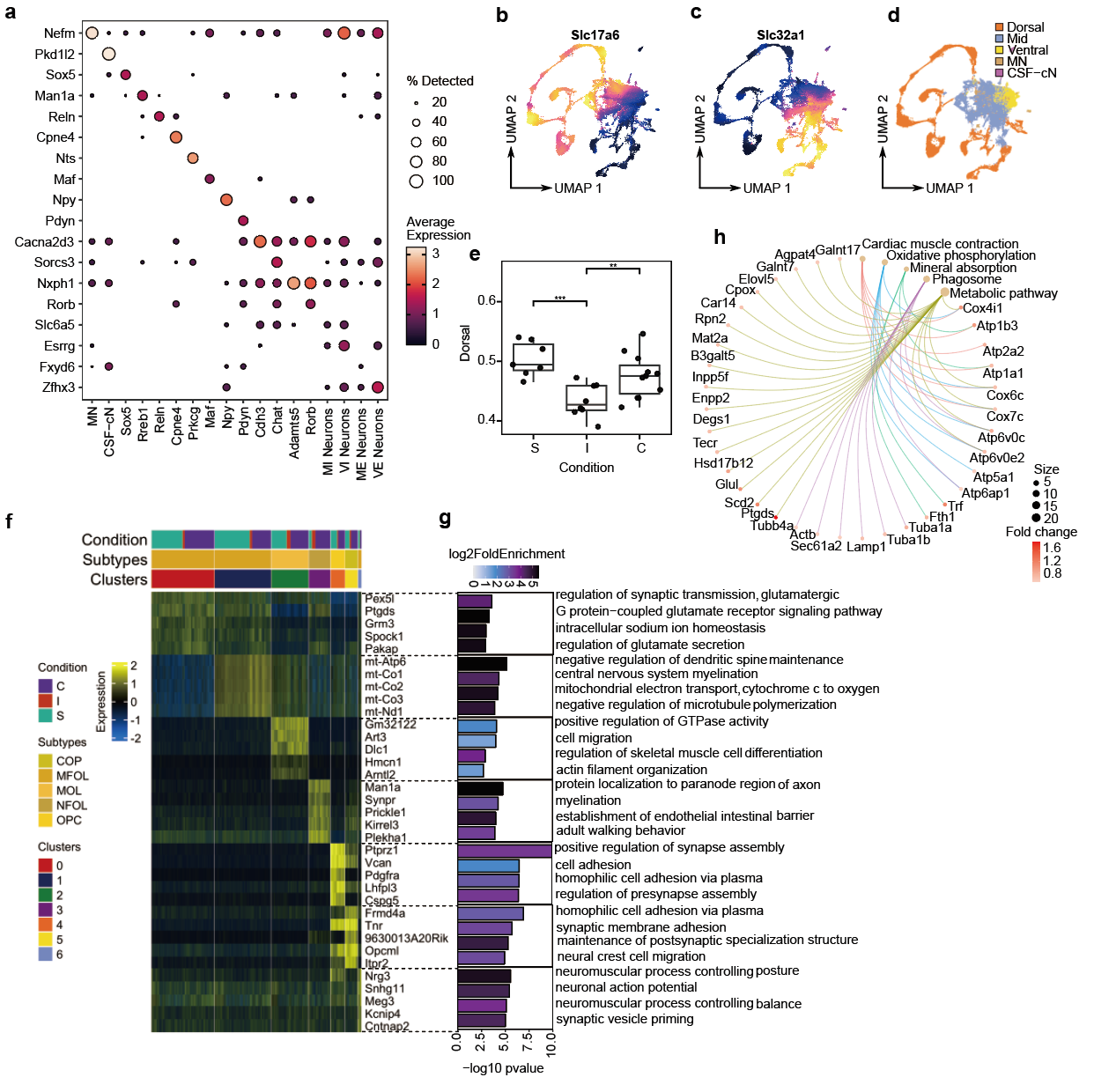


**Figure S24. Annotation and heterogeneity of neuron and oligodendrocytes different conditions. a.** Dot plot showed the main markers for each subtype of neuron in snRNA-seq. **b.** UMAP plot of expression of key neurotransmitters for excitatory (Slc17a6) in neuron in snATAC-seq. **c.** UMAP plot of expression of key neurotransmitters for inhibitory (Slc31a1) in neuron in snATAC-seq. **d.** UMAP plot of subtypes of neuron in snATAC-seq, color by location; location was assigned based on in situ hybridization validation experiments in public dataset. **e.** Boxplot showed the composition of dorsal neurons of snATAC-seq across different condition. Abundances of dorsal neurons in S(Sham) and C(therapied via Ce@UCNP-BCH after spinal cord injury) were compared with their abundances in I(injured) with two-side Wilcoxon testing and Bonferroni correction for multiple comparisons, and the symbolic number coding of adjusted P values are listed in the plots. The boxplots were constructed with data from 7 S samples, 8 I samples and 11 C samples. **f.** Complex heatmap with additional information of main cell type and condition showing the top markers for each subtype of snRNA-seq. **g.** GO term enrichments for the highly expressed genes in each cluster from f. FDR <= 0.01 & Log2FC >= 0.5 & ontology was biological process; only plot the top four terms. **h**. Network plot showed the most significant results of gene set enrichment analysis for highly expressed genes of MFOLs in the group of C comparing to I(injured).


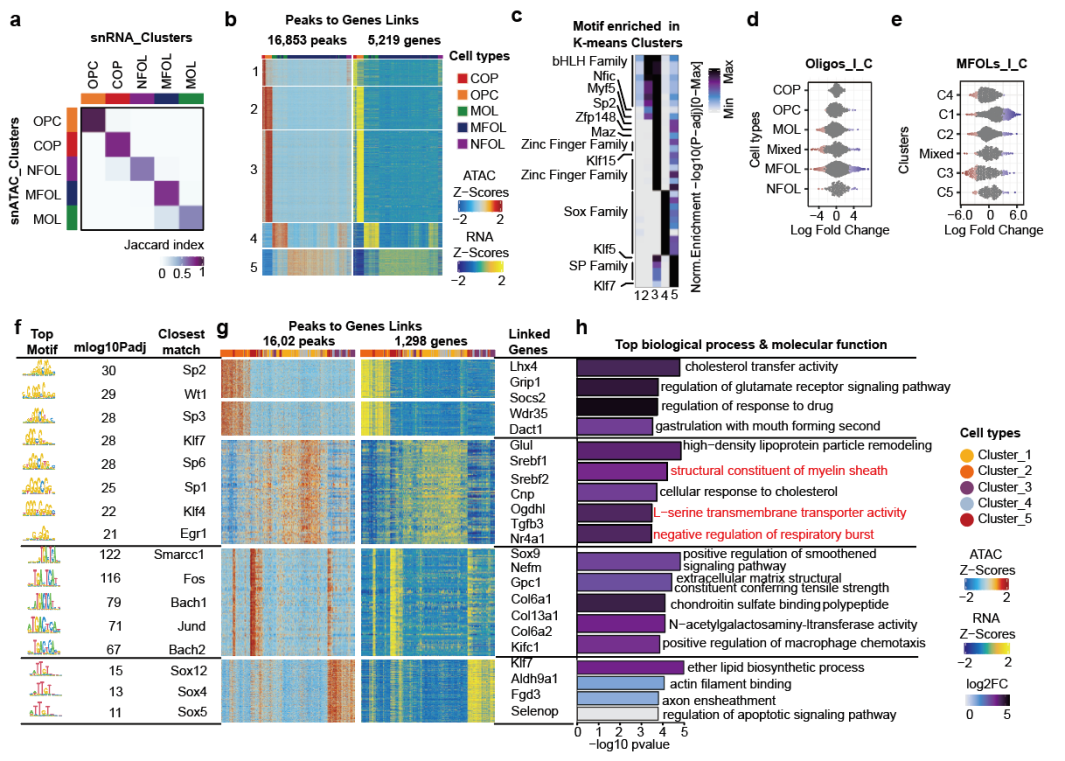


**Figure S25. Integration of snATAC-seq and snRNA-seq uncovers the heterogeneity of oligodendrocytes. a.** Correspondence between snATAC-seq and snRNA-seq cluster label in oligodendrocytes. **b.** The heatmap showing the chromatin accessibility on the left and gene expression on the right for the 23,452 peak-to-gene linkages. The peak-to-gene linkages were clustered using k-means clustering (K=5, same as cell types). **c.** Hypergeonomics enrichment p-values of TF motif in peaks from each K-means clusters from h. **d.** Plot visualized the distribution of differential abundance between condition I(injured) and condition C(therapy) in different cell types for oligodendrocytes in snATAC-seq using MiloR. Cell type fraction less than 0.7 were defined mixed. Positive values indicated that the cell types were enrich in the condition C(therapy). **e.** Plot visualized the distribution of differential abundance between condition I(injured) and condition C(therapy) in different sub cluster for MFOLs in snATAC-seq using MiloR. Cell type fraction less than 0.6 were defined mixed. Positive values indicated that the cell types were enrich in the condition C(therapy). **f.** The table lists top motifs identified, along with their mlog10Padj values. **g.** The heatmap showing the chromatin accessibility on the left and gene expression on the right for the 1,973 peak-to-gene linkages in MFOLs. The peak-to-gene linkages were clustered using k-means clustering (K=5, same as sub cluster). Closest matching transcription factors shown on the left, linked genes shown on the right. **h.** GO term enrichments for the highly expressed genes in each k-means clusters from k. Ontology was biological process and molecular function; only plot the top terms.


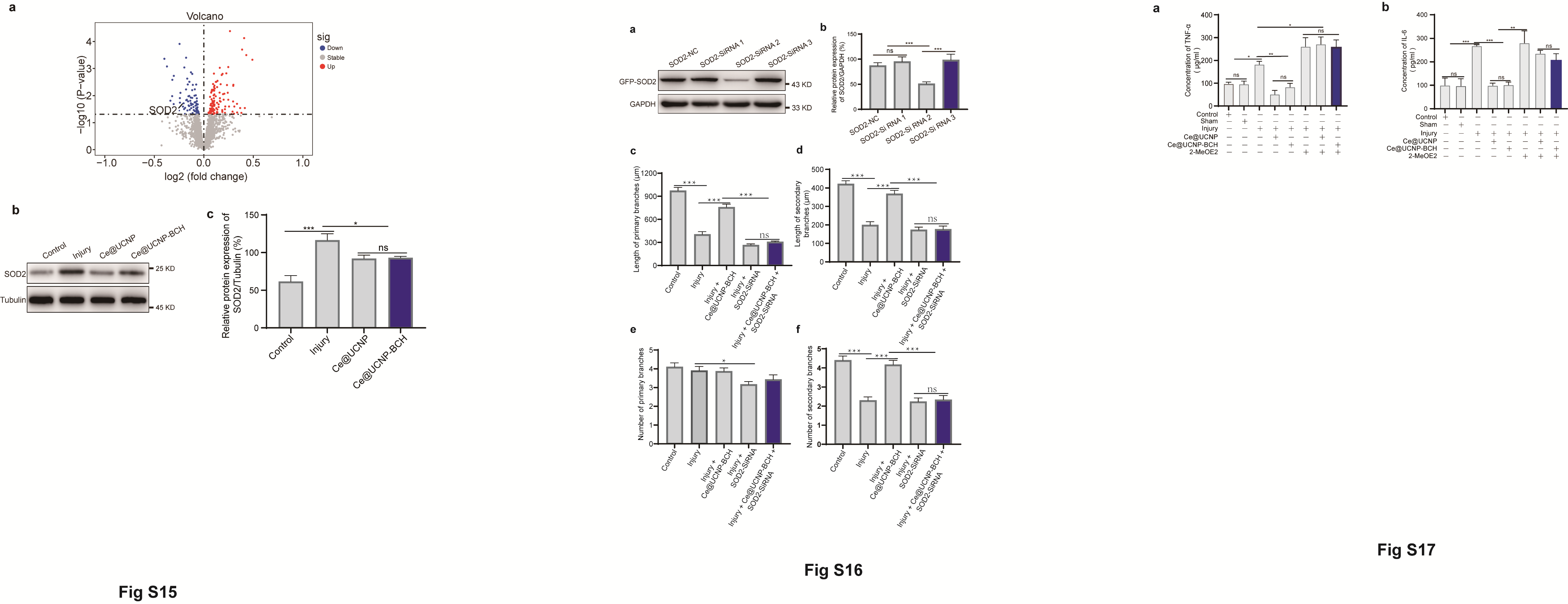
**Figure S26. Mechanism of the** **therapeutic effect of Ce@UCNP-BCH. a**. Volcano map showing the different expressed proteins of Ce@UCNP-BCH targets. **b**. Western blot analysis of SOD2 expression levels in mouse cortical neurons in the control, injury, injury + Ce@UCNP and injury+ Ce@UCNP-BCH group. **c**. Densitometric quantifications of western blots analysis of SOD2 expression levels in mouse cortical neurons in the control, sham, injury, injury + Ce@UCNP and injury+ Ce@UCNP-BCH group (n=9). * means p < 0.05, ** means p < 0.01, *** means p < 0.001, ns means not significant.


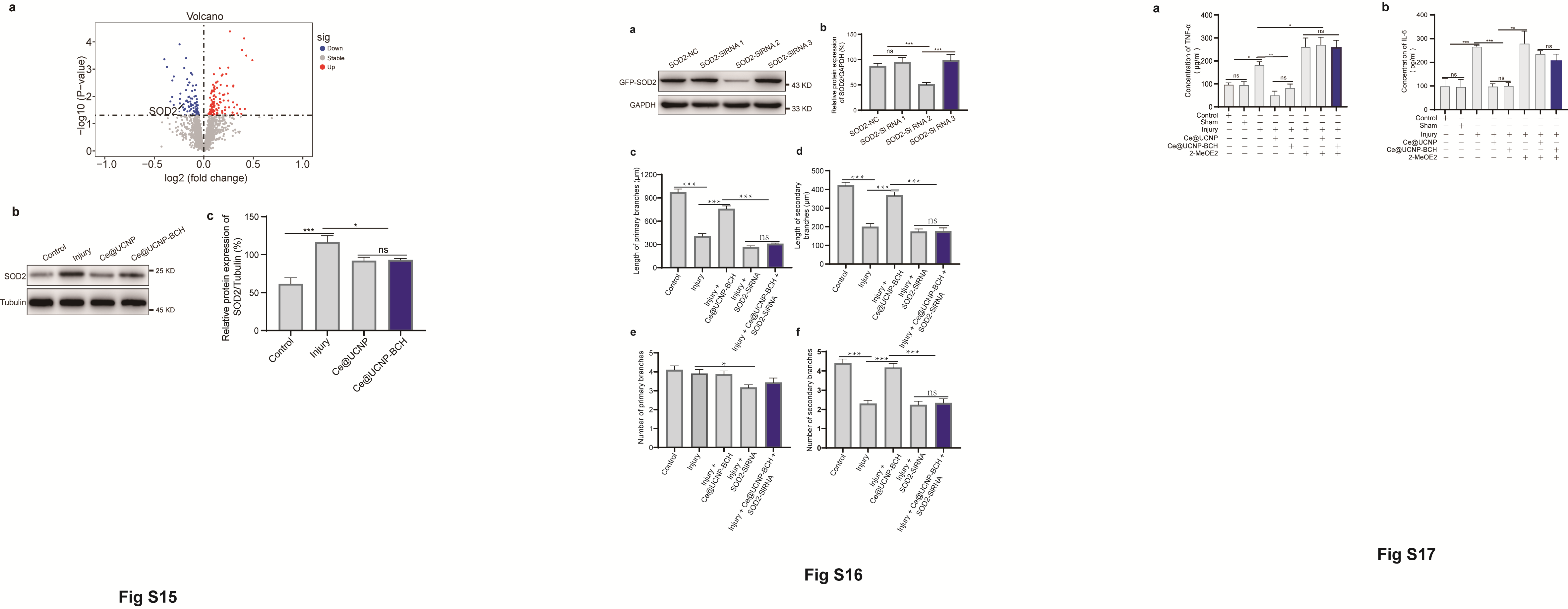


**Figure S27. The interference efficacy of SOD 2-SiRNA in the therapeutic effects of Ce@UCNP-BCH. a.** Expression levels of SOD2 and GAPDH were detected using Western blotting for lysates from 293T cells treated with SOD 2-SiRNA. **b.** Densitometric quantifications of western blots depicted in **a** (n=3). Quantified relative length of neuronal primary **c** and second **d** branches, and number of neuronal primary **e** and second **f** branches in control, injury, injury + Ce@UCNP-BCH, injury + SOD2-SiRNA group, injury + Ce@UCNP-BCH + SOD2-SiRNA group (n=30). * means p < 0.05, ** means p < 0.01, *** means p < 0.001, ns means not significant.


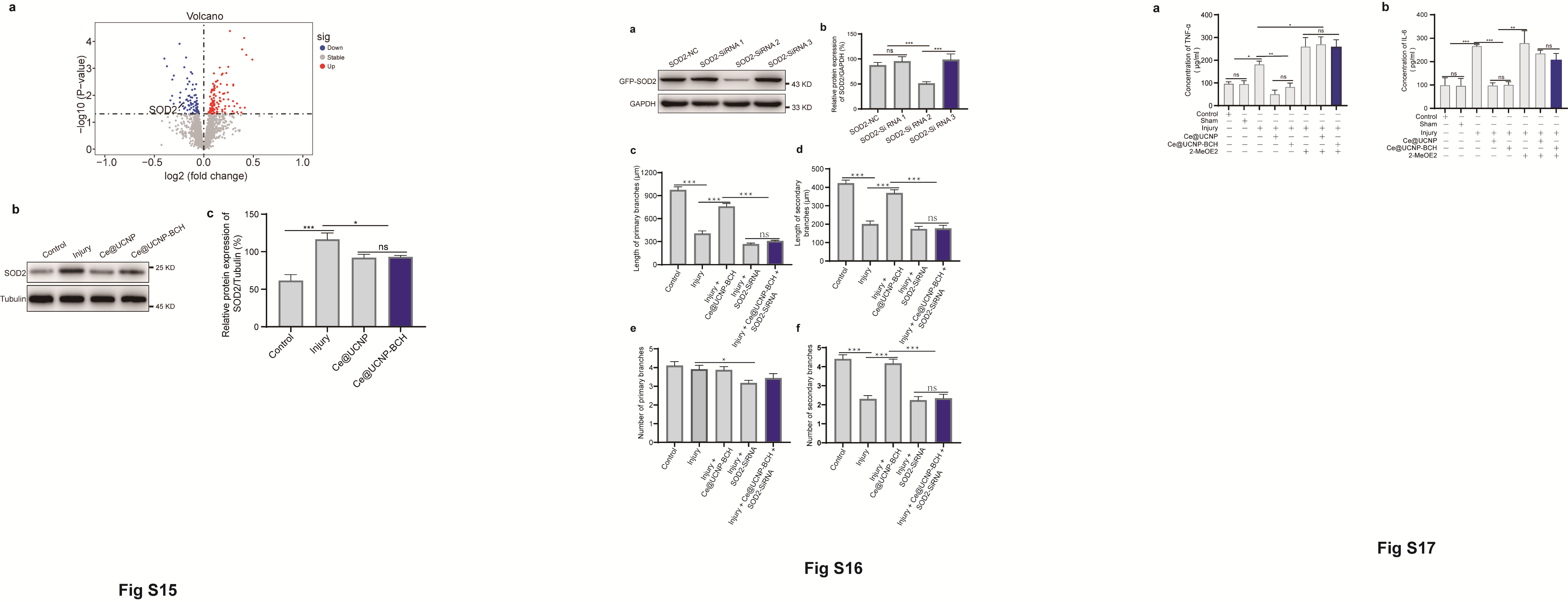


**Figure S28. ELISA analysis of the concentration of TNF-α a and IL-6 b in serum of mouse in control, sham, injury, injury + Ce@UCNP, injury + Ce@UCNP-BCH, injury + 2-MeOE2, injury + Ce@UCNP + 2-MeOE2, injury + Ce@UCNP-BCH + 2-MeOE2 group** (n=3)**.** * means p < 0.05, ** means p < 0.01, *** means p < 0.001, ns means not significant.
